# Cost of Delivering Health Care Services in Public Sector Primary and Community Health Centres in North India

**DOI:** 10.1371/journal.pone.0160986

**Published:** 2016-08-18

**Authors:** Shankar Prinja, Aditi Gupta, Ramesh Verma, Pankaj Bahuguna, Dinesh Kumar, Manmeet Kaur, Rajesh Kumar

**Affiliations:** 1 School of Public Health, Post Graduate Institute of Medical Education and Research, Chandigarh, India; 2 Department of Community Medicine, Pt. BD Sharma Post Graduate Institute of Medical Sciences, Rohtak, Haryana, India; 3 Department of Community Medicine, Dr. Rajendra Prasad Government Medical College, Kangra, Himachal Pradesh, India; UNAIDS, GUYANA

## Abstract

**Background:**

With the commitment of the national government to provide universal healthcare at cheap and affordable prices in India, public healthcare services are being strengthened in India. However, there is dearth of cost data for provision of health services through public system like primary & community health centres. In this study, we aim to bridge this gap in evidence by assessing the total annual and per capita cost of delivering the package of health services at PHC and CHC level. Secondly, we determined the per capita cost of delivering specific health services like cost per antenatal care visit, per institutional delivery, per outpatient consultation, per bed-day hospitalization etc.

**Methods:**

We undertook economic costing of fourteen public health facilities (seven PHCs and CHCs each) in three North-Indian states viz., Haryana, Himachal Pradesh and Punjab. Bottom-up costing method was adopted for collection of data on all resources spent on delivery of health services in selected health facilities. Analysis was undertaken using a health system perspective. The joint costs like human resource, capital, and equipment were apportioned as per the time value spent on a particular service. Capital costs were discounted and annualized over the estimated life of the item. Mean annual costs and unit costs were estimated along with their 95% confidence intervals using bootstrap methodology.

**Results:**

The overall annual cost of delivering services through public sector primary and community health facilities in three states of north India were INR 8.8 million (95% CI: 7,365,630–10,294,065) and INR 26.9 million (95% CI: 22,225,159.3–32,290,099.6), respectively. Human resources accounted for more than 50% of the overall costs at both the level of PHCs and CHCs. Per capita per year costs for provision of complete package of preventive, curative and promotive services at PHC and CHC were INR 170.8 (95% CI: 131.6–208.3) and INR162.1 (95% CI: 112–219.1), respectively.

**Conclusion:**

The study estimates can be used for financial planning of scaling up of similar health services in the urban areas under the aegis of National Health Mission. The estimates would be also useful in undertaking equity analysis and full economic evaluations of the health systems.

## Introduction

Indian healthcare delivery system comprises of 152,326 sub-centres (SCs), 25020 primary health centres (PHCs), 5363 community health centres (CHCs), 1024 sub-district hospitals and 755 district hospitals [1]. The sub-centres being the most peripheral units of health care delivery caters mainly to preventive and promotive care with some curative services for minor ailments such as fever, acute respiratory illnesses, diarrhoea etc being provided by auxiliary nurse midwives (ANM) and community health workers (CHW). PHCs are referral centres for sub-centres and are first contact point between community and the qualified medical doctors in India. As per Indian Public Health Standards (IPHS), a PHC caters to a population of around 20,000 in hilly, tribal and desert areas while 30,000 in better accessible plain areas [2]. It consists of medical officers, staff nurses, health supervisors like lady health workers, head staff nurse and supporting staff to provide outpatient and inpatient care [2].

Patients who require further specialist care are referred to next higher level of heath service delivery called CHCs which cater to a population of around 80,000–100,000 [3]. These are designed to be equipped with at least four specialists in the areas of medicine, surgery, pediatrics and gynecology along with the complementary medical and para medical staff with facilities for 30 indoor beds; operation theatre, labour room, X-ray machine, pathological laboratory etc [3].

The extent of utilization of primary health care centers for antenatal care services among the public health facilities in India is 22% [4]. Nine percent of total institutional deliveries, i.e. using a health facility with all the essential life saving amenities for giving birth to a child under the supervision of competent health personnel and skilled birth attendant, happens at the level of PHCs and 7% at CHCs [4]. In terms of total public sector spending for healthcare in India, 41% is spent on primary health care and 15% on secondary healthcare [5]. While some primary care is also provided by the secondary and tertiary care institutions, however, the extent of primary care provision in these two categories is relatively less. Moreover, nearly one-fifth (18.25%) of all health care cost is constituted by the outpatient care provided through PHCs, dispensaries i.e. health care facilities for the out-patient care where medical care and medicines are dispensed and sub-centers [5]. These facts suggest that there is a significant volume of service provision at the level of PHCs & CHCs.

Moreover, at national level, there has been an increase of 6300 sub-centers, 1784 PHCs and 2017 CHCs in 2014 as compared to those existing in year 2005, implying a 7.7% and 60.3% increase in the number of PHCs (from 23236 to 25020) and CHCs (from 3346 to 5363) respectively since the introduction of National Rural Health Mission (NRHM) in the country [1]. There has also been a significant increase in the number of manpower positioned in these health facilities in the last decade with an increase of 63%, 35% and 15% in the numbers of ANMs, allopathic doctors at PHCs and specialist doctors at CHCs respectively. These facts highlight that considerable amount of resources are spent at the level of PHCs and CHCs [6]. Now, with the advent of National Urban Health Mission, health care delivery structure similar on the lines of rural areas is being developed in urban India. So, there is a need for evidence generation for the effective planning and allocation of resources for a large scale up [7].

Also, there is limited availability of literature on costs spent per service delivery at level of primary and community health centers and the present literature is more than a decade old which limits its application [8]. Most of the health costing studies in India highlight the cost of delivering particular services like pediatric care [9], referral transport [10], newborn care in district hospitals [11], specific diseases like respiratory diseases [12] or typhoid [13] and service provider like at primary health center [14] or district hospital [15].

With the commitment of Government to provide each of its citizen with universal health care, it is important from the perspective of planners and policy makers as to how much cost is being levied by the government per unit service delivered. This can also be used in terms of equity research, i.e. benefit incidence analysis, and determining allocative efficiency of Government health care services. In this paper, we reported the overall annual cost for delivering the gamut of services at PHC and CHC level in public sector. Secondly, we assessed unit cost of specific services delivered at PHCs and CHCs.

## Methods

### Study settings and service platform

This study was undertaken in three states of north India namely, Haryana, Punjab and Himachal Pradesh. Together, these states comprise around 60 million of Indian population. Multistage stratified random sampling was followed to select the districts and health facilities for this study. States of Haryana, Punjab and Himachal Pradesh were chosen purposively as there was specific policy requirement for estimating cost of health care services in these states. In the first stage, 10% of districts were chosen randomly from each state (two of 21 districts in Haryana, two of 22 districts in Punjab and one out of 12 districts in Himachal Pradesh). Secondly, within districts, one community health center from each district was selected. Later one more CHC were added each from Punjab and Haryana to capture their relatively bigger population than Himachal Pradesh. Then 10% of primary health centers were selected by process of simple random sampling (lottery method). Punjab has a different system of public health facilities than Haryana and H.P. In case of Punjab state, a large number of PHCs were upgraded as Block PHCs which had infrastructure resembling to that of a CHC. Similarly, a number of new health facilities–Mini PHCs were created which were managed by the Local Governments. Hence, the numbers of PHCs which resemble the actual norms of a typical PHC were relatively less, resulting in a smaller sampling frame, ultimately resulting in relatively lesser number of overall PHCs drawn from the state. All of these facilities were completely government funded.

As per IPHS, a PHC and CHC should provide out-patient services, in-patient services, 24-hour emergency services and referral services. It also provides reproductive and child health care services like antenatal care, institutional deliveries, postpartum care, immunization, child health care etc [2, 3]. Along with MCH services, they also take care of family planning, adolescent health, school health, nutritional programmes, promotion of safe drinking water and sanitation, disease surveillance and control of epidemics, collection of vital events and behavior change communication activities. Besides, the services mentioned, the staff is also involved in the record keeping and attending review and monthly meetings. So the study centers were evaluated for all the above mentioned services. The details of the services being provided at the two levels is described in S1 and S2 Tables.

### Data collection

Economic cost of services was assessed from the health system perspective, using bottom up costing methods. The data was collected during April to June 2013 by field investigators who were qualified up to post graduation and were trained for collecting data on costing. Data on both capital and recurrent resources spent for delivering health services during last one year (April 2012 to March 2013) were collected (S1 and S2 Tools). The capital resources comprised of building, equipment and other non-consumables which lasted for a period of more than one year. Trainings which were not likely to be repeated within one year period were also treated as capital costs. Recurrent costs comprised of the staff salaries, drugs, consumables and overheads such as water, electricity bills etc. Apart from this, data on incentives paid under various health schemes and other annual maintenance grants were also collected.

Data were collected through review of various records, registers, reports, interview of key stakeholders and facility observations. For example, routine records like outpatient registers, inpatient registers and monthly reports were used to collect the number of services provided during the year. Stock registers were used to enlist the quantity of various drugs and consumables consumed during the reference period, along with equipment and non-consumable items present and used. This was supplemented with data on incentives paid under various schemes (for instance cash transfers under *Janani Suraksha Yojana* scheme etc; untied funds and annual maintenance grants which were collected from the office of Civil Surgeon of the respective districts.

Facility surveys were undertaken to assess the capital and physical infrastructure such as building, space, furniture and other equipments present in the health facilities. The infrastructure details were ascertained room wise along with the purpose for which they were being used. All the staff members were interviewed with semi structured interview schedule on time allocation for different services in the last one week. Interviews included information on frequency of the activities like daily, weekly, fortnightly, monthly, quarterly, semi annually or annually; the time spent per activity and the total number of patients seen on the day of activity. The less frequent activities, for instance immunization days, pulse polio days, trainings, etc were performed for whole day. Thus, the complete day was allocated to the same activity. Time spent by staff members on the administrative work was also collected in the time allocation sheets. All respondents were interviewed after obtaining written informed consent. The data collection tools for PHC and CHC are attached as S1 and S2 Tools. The estimates of the time spent by each staff on various activities were also supplemented with observations on time spent on daily activities during the period of data collection.

### Data analysis

Costs of capital resources were annualized by considering the life of the capital item. A discount rate of 5% was applied in accordance with the guidelines given by the International society for Pharmacoeconomics and Outcome Research for India [16]. Capital resources such as building and space were estimated as the floor area in square feet. The information about the quantity of resources was obtained from stock registers, accounts records of District Health Office and health facility surveys. The opportunity costs of capital resources like land and building were estimated by interviewing key informants to obtain the prevalent market rental price. For furniture and equipments, standard literature on the life of capital items was reviewed [17, 18]. The local staff at the health facility was also interviewed to know their perceptions on the same. The costs of equipments were also obtained from local distributors and from internet search of relevant websites [19]. The costs of all recurrent resources like drugs, consumables etc were obtained from the rate contract list of state governments estimated by applying the price to the quantity of resources consumed. Average prevailing market prices for the year 2014–2015 were used. All the prices were then converted to the price in the year 2012–2013 using prevailing gross domestic product deflators [20].

Certain resources in the facility were used solely for one activity while the others for instance, furniture, equipment and room space were used in more than one activity. If the resources were directed for one service, then the complete cost was allocated to that service only but if any resource is utilized jointly in two or more services then it was apportioned among those services using appropriate statistics. For example, if the capital cost of the room (or equipment or medicine or furniture) was shared among two or more services/programs, it was apportioned by the proportion of time it was used for a particular service or program. This indicator of apportionment combined the effect of the number of clients for a particular service and time spent on each client for that service. The costing assumptions are mentioned in detail in S3 Table.

The overall costs of health services provided at primary and community health centers were presented as inpatient, outreach and outpatient services; promotive, preventive, curative and indirect administrative services. The overall costs for human resource, capital, consumables, equipment, drugs, overheads, IEC were also estimated for each of the functional group of services. Along with the overall annual costs at health facilities, per capita unit cost of service provision per year was also computed. Besides these, per capita unit costs per specific services provided were calculated like for antenatal care, institutional deliveries & post natal care, immunization and family planning services. They were also calculated for curative services used i.e. per outpatient consultation or per bed day hospitalization etc. It was computed by combining the value of all the resources spent on provision of care during a year and dividing it by the total clients who used the service in respective facility in that year.

The sample was simulated 999 times using the bootstrap method. SPSS 21 was utilized for analyzing the data. The mean estimates were calculated for unit costs along with its 95% confidence limits.

### Sensitivity analysis

We undertook a univariate sensitivity analysis wherein the base value of salaries, price of equipment, building cost, rental prices and assumptions on time allocation were varied by 25% on both sides. Prices of drugs and consumables show wide variation; hence we varied these by 90% on lower limit to 100% on upper limit. We also estimated the sensitivity of the annual cost and unit cost for providing overall services to variations in discount rates i.e. at 3% and 10% [16].

## Results

### Profile of study centers

A total of fourteen primary and community health centers were studied in three states of north India–Haryana, Himachal Pradesh and Punjab. The average population covered by a PHC and CHC is the study sample was 37,635 and 147,941 respectively, while the number of beds was as per norms i.e. 6 for PHC and 30 for CHC (Ref. Table 1). The ratio of doctor to nurse and doctors to bed varied from 1:1 and 1:3 respectively at PHC level, and from 1.7:1 and 1:4 respectively at CHC level. In terms of volume of services provided, a CHC catered a 2.5 times and 8.2 times the number of outpatients and hospitalization as compared to a PHC respectively. The annual institutional deliveries varied from 106 at PHC to 577 at CHC level.

**Table 1:** Profile of study centers.

Characteristic	Mean (Range)	
	PHC (n = 7)	CHC (n = 7)
Population covered	37,635 (25,729–57,918)	147,941(125,000–254,943)
Number of Beds	6	30
Human Resources	15 (10–26)	42 (23–60)
Doctors-Nurses ratio	1:1	1.7:1
Doctors-beds ratio	1:3	1:4
Outpatient attendance	25,958 (14,259–62,923)	64,661 (35,248–137,646)
Institutional Deliveries per year	106 (0–933)	577 (332–1282)
Hospitalizations	387 (0–171)	3195 (534–8779)

### Annual costs

**Fig 1:**
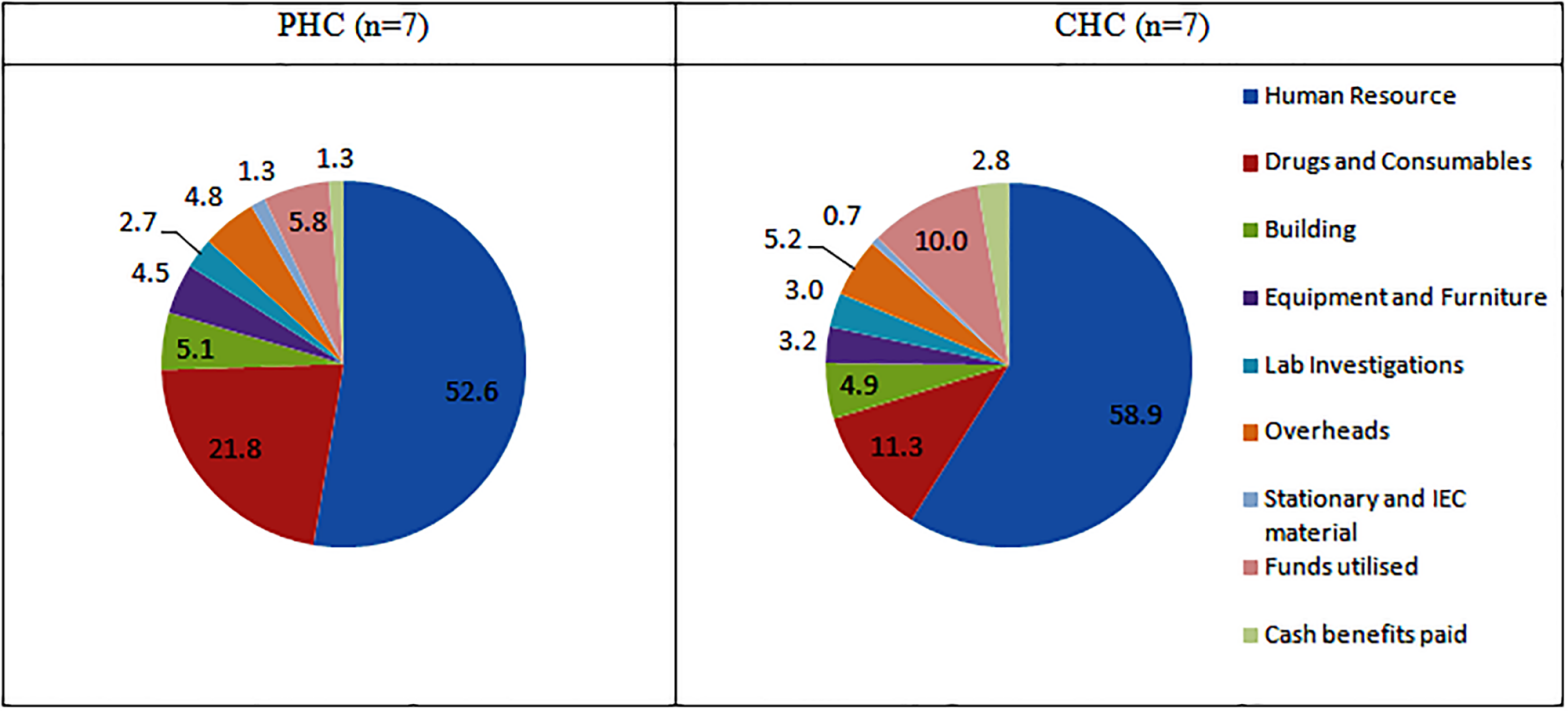
Proportional distribution of cost of various components of health service delivery at PHCs and CHCs in north India.

The mean annual costs for providing health services at PHCs and CHCs were INR 8.8 million (95% CI: 7,365,630–10,294,065) and INR 26.9 million (95% CI: 22,225,159.3–32,290,099.6) respectively. Table 2 provides the annual mean costs of various components of health service delivery at primary and community health centers. Cost of human resource alone accounted for 52.6% and 58.9% of total cost at PHC and CHC respectively. The proportional cost of providing drugs and consumables was 21.8% at PHC and 11.3% at CHC. (Ref Table 2, Fig 1)

**Table 2:** Annual costs of delivering health care services at Primary and Community Health centers in north India.

Annual Cost	PHC(n = 7)			CHC(n = 7)		
	Mean Cost (INR)	95% Confidence interval		Mean (INR)	95% Confidence Interval	
		Lower limit	Upper limit		Lower limit	Upper limit
Human Resource	4,744,379	3,476,203	6,307,073	15,866,007	13,578,670	17,885,743
Drugs	1,704,385	994,367	2,526,073	2,496,358	927,868	4,295,269
Equipment	247,282	148,652	352,471	565,052	404,688	754,073
Consumables	264,583	167,970	346,540	549,970	225,287	1,017,283
Capital	457,188	281,941	660,228	1,328,387	880,384	1,875,420
Furniture	154,716	83,307	232,584	289,995	209,681	376,852
Lab investigations	244,834	59,643	446,964	815,065	447,783	1,200,162
Overheads	436,967	229,080	669,263	1,391,977	627,172	2,642,428
Stationary	80,127	10,303	189,155	150,259	31,765	297,474
IEC material	34,413	20,219	55,883	34,452	20,731	43,939
Funds utilized	525,004	226,601	825,106	2,685,085	1,393,984	4,061,792
Cash benefits paid	117,672	73,429	165,743	744,474	503,954	979,520
Total Annual Cost	8,858,520	7,365,630	10,294,065	26,917,082	22,225,159	32,290,100

**Fig 2:**
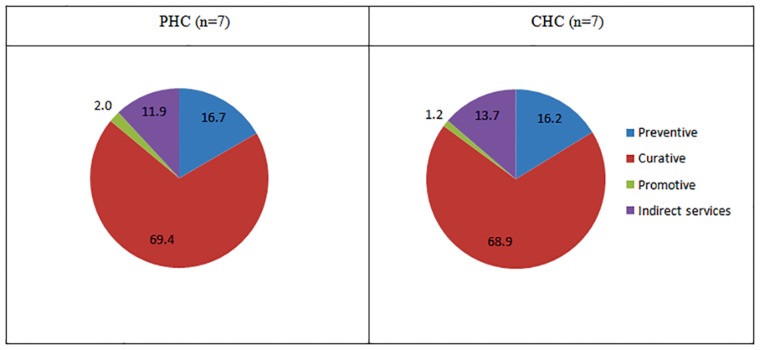
Proportional distribution of cost of health service delivery by the level of services at PHCs and CHCs level in north India.

**Fig 3:**
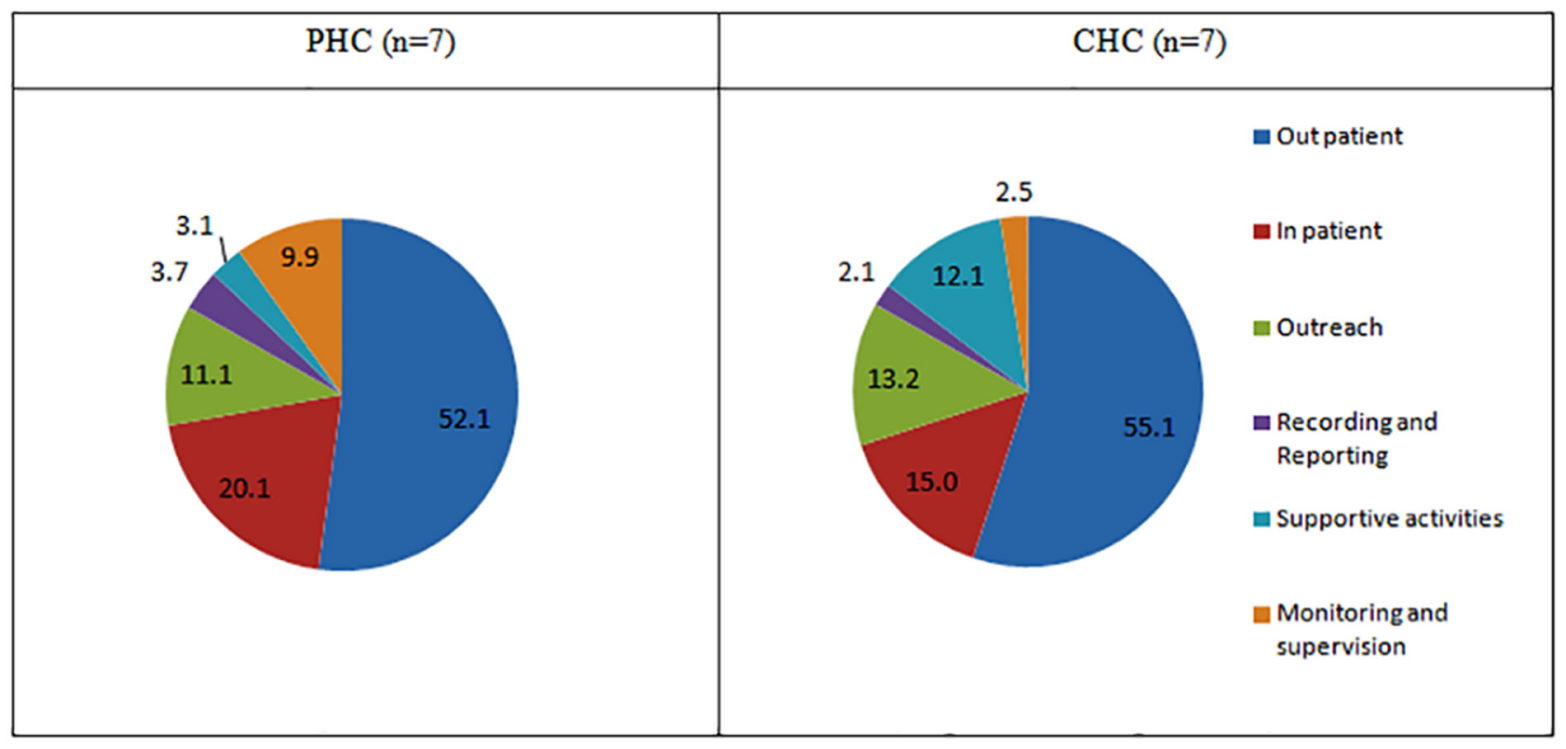
Proportional distribution of cost of health service by nature of service provided at PHCs and CHCs in north India.

Fig 2 shows that 69.4% and 68.9% of the total cost incurred for provision of services at PHC and CHC respectively was on account of curative services followed by preventive services (16–17% in both PHCs and CHCs). Fifty two percent and fifty five percent of total costs were spent for outpatient consultations followed by 20% and 15% on in-patient treatment at PHCs and CHCs respectively. (Refer Fig 3)

### Unit costs

Unit costs per service provided were estimated as the ratio of total annual costs for the particular service and total number of beneficiaries in one year period. It was found that provision of services at PHC and CHC incurred a cost of INR 170.8 (95% CI: 131.6–208.3) and INR162.1 (95% CI: 112–219.1) per capita per year respectively to provide a complete package of preventive, curative and promotive services (Ref Table 3). Average costs of providing one full antenatal care to a pregnant woman at PHC and CHC were INR 677.2 (95% CI: 520.0–834.5) and INR 649 (95% CI: 363.8–998.1) respectively. The costs incurred per outpatient consultation at PHC and CHC were INR 139.0 (95% CI: 109.1–171.7) and 172.3 (95% CI: 126.3–229.1) respectively while unit costs per bed day during hospitalization were INR 690.6 (95% CI: 382.6–1025.4) at PHC and INR 687 (95% CI: 224.4–1494) at CHC. Table 3 lists unit costs per service delivery per year for provision of various health services at the mentioned health facilities. The stratified estimates on cost per service delivered for PHCs and CHCs covered under study are given in detail in Tables 4 and 5 respectively.

**Table 3:** Unit costs of delivering health care services at Primary and Community Health Centers in north India.

Unit Cost	PHC (n = 7)			CHC (n = 7)		
	Mean INR	95% Confidence Interval		Mean INR	95% Confidence Interval	
		Lower Limit	Upper Limit		Lower Limit	Upper Limit
Per capita for overall service	170.8	131.6	208.3	162.1	112.0	219.1
Per outpatient consultation	139.0	109.1	171.7	172.3	126.3	229.1
Per bed day hospitalization	690.6	382.6	1,025.4	687.1	224.4	1,494.0
Per antenatal care*	677.2	520.0	834.5	649.0	363.8	998.1
Per institutional delivery*	2,039.2	1,547.9	2,702.1	2,225.8	1,289.2	3,518.2
Per postnatal care	740.4	497.9	1,003.8	705.7	574.3	837.2
Per child immunized	82.0	41.8	145.2	116.1	53.6	196.8
Per newborn corner case	2,180.3	456.4	3,779.5	2,495.0	167.1	5,592.1
Per IUCD procedure	180.7	116.1	262.9	77.9	53.1	114.7

*One outlier observation in case of cost of Antenatal care and Institutional delivery was excluded from the bootstrap analysis.

**Table 4:** Cost of delivering health care services in seven Primary Health Centers from north India.

Facility Id	P1	P2	P3	P4	P5	P6	P7
Total annual cost	10,053,313	12,003,494	10,797,503	8,842,610	7,094,026	6,024,504	7,194,191
Per capita unit cost	141.5	245.7	132.2	200.8	91.0	155.7	228.8
Per outpatient consultation	83.6	152.3	82.9	219.1	156.2	136.8	142.2
Per bed day hospitalization	329.6	1,195.1	193.7	1,002.3	732.4	NA*	NA*
Per antenatal care	500.2	657.7	712.9	787.8	941.9	443.4	3,789.5
Per institutional delivery	2,127.8	1,590.1	1,831.2	3,282.7	1,364.1	NA*	35,503.5
Per postnatal care	1,238.4	459.0	540.2	1,257.3	275.8	670.9	741.0
Per child immunized	203.0	156.4	58.6	66.0	30.2	52.4	1392.7
Per IUCD procedure	153.0	1,632.2	85.8	312.3	127.9	224.6	328.6
Per Newborn care corner case	1,107.2	239.5	2,704.2	4,670.2	NA*	NA*	NA*

*NA = when the utilization of the service was nil or zero

**Table 5:** Cost of delivering healthcare services in seven Community Health Centers from north India.

Facility Id	C1	C2	C3	C4	C5	C6	C7
Total annual cost	41,362,572	26,016,884	19,604,148	19,476,110	25,509,871	28,270,103	28,179,886
Per capita unit cost	111.9	95.2	110.2	285.9	147.4	113.8	270.5
Per outpatient consultation	101.3	92.5	198.2	197.5	197.2	307.6	112.1
Per bed day hospitalization	277.0	134.6	2,998.4	551.2	216.4	418.2	214.0
Per antenatal care	616.0	63.6	471.1	1,263.9	1,267.1	260.8	600.7
Per institutional delivery	778.0	1,923.7	5,417.2	1,820.3	1,865.9	2,868.8	906.3
Per postnatal care	751.4	529.1	428.4	616.4	939.0	830.3	845.6
Per child immunized	160.4	39.4	28.4	186.1	83.0	19.0	296.9
Per IUCD procedure	85.9	36.1	67.0	160.3	71.5	46.7	8,056.4
Per Newborn care corner case	93.3	NA*	3,970.8	NA*	175.9	7,938.0	296.8

*NA = when the utilization of the service was nil or zero

### Sensitivity analysis

There was not much difference in the annual and unit costs with the change in discount rates. The average annual cost of providing overall range of healthcare services at PHC varies from INR 8,825,410(95%CI: 7,335,972.1–10,245,097) to 8,902,439 (CI: 7,400,476.6–10,336,540) and at CHC, it varies from INR 26,865,435(95% CI: 22,531,211.5–32,386,335.4) to 27,053,237 (95% CI: 22,721,201.8–32,613,788.6) on varying discount rate from 3% to 10% respectively. Similarly, the unit cost per capita changed minimal i.e. from INR 170.2 (95% CI: 131.2–207.8) to 171.6 (CI: 132.5–209.1) at PHC and from INR 161.8(CI: 110.2–212.1) to 163.0 (CI: 110.8–213.5) at CHC on varying the discount rates from 3% to 10% respectively [16].

The sensitivity analysis to see the effect of variation in input costs on annual cost at CHCs and PHCs showed that the annual costs at PHCs were most sensitive to drugs (70.5%) followed by salaries paid to staff (28.3%) while at CHCs, human resource (63%) affected the annual cost most followed by drugs and consumables (33.6%). (Refer S1 and S2 Figs)

## Discussion

Primary and community health centres in public health sector provide health care services to a large proportion of population in India. Detailed analysis of cost of provision of primary health care services through community health workers in sub-centres and primary health centres is available [8, 21]. However, the evidence base for cost of provision of health care through community health services is very weak. We undertook this study in fourteen health centers in three states of north India to generate evidence on cost of health services provided at the public sector primary and community health centers.

To our knowledge, this is the first comprehensive study to assess the cost of primary health care services at PHCs and CHCs covering facilities from three states. We used standardized costing methodology and took data for one complete year to exclude the seasonal variation of diseases and service utilization. Standard method for analyses was used to present detailed costing of services provided. In our analyses, we found that the unit costs of providing an entire range of preventive, curative and promotive care were INR 170.8 at PHC and INR 162 at CHC per capita per year. Salaries constitute more than half of the total cost of service delivery at both the levels. Unit costs per specific services were INR 677.2 and INR 649 per full ANC provided; INR 740.4 and INR 705.7 per postnatal care; INR 180.7 and INR 77.9 per IUCD insertion procedure at PHC and CHC respectively. Around two third of the costs were incurred on curative services, while less than 2% were spent on promotive health services.

Evidence from couple of previous studies were either too old or rely on only one health facility data to determine cost. For example, Anand et al. estimated cost of a single primary health care center in New Delhi in 1993 [8]. The findings of our study are comparable to those reported in that study. Salaries constituted 62% of the total annual cost incurred on a PHC while in our study the proportion was 52.6%. The total operational annual cost for PHC was INR 777,015, with a unit per person per year cost of INR 30 while in our study these values were higher. The difference in reported costs could be attributed to the time difference of two decades between both studies and it is a possibility that the inflation might have changed the currency values many folds between two time frames. Discounting for the time difference and adjusting for inflation, unit cost of service comes out to be INR 95.10, which is far less than our estimate of INR 170.8 at PHC. Relatively higher unit cost of services at PHCs in our study, even after adjusting for inflation, could be as a result of relatively higher inflation of health care costs as compared to general inflation rate.

Mathur et al estimated the cost of providing curative services at three primary care centers in a city in Gujarat in year 2010 [14]. The unit cost of curative care services in this study ranged from INR 29.43 to 88.26 in PHCs while the total annual cost for running PHC ranged from INR 385,668–612,422. Several factors could explain difference in annual costs between this and our study. Firstly, there is a difference in the population covered by health facilities in both the studies. While in both the above mentioned compared studies, a primary care center covered 25,000–30,000 population, PHCs in our study covered a range of population varying from 25,729 to 57,918. Secondly, inflation of health care expenditures, as explained above, could justify the differences. Finally, with the introduction of NRHM, more finances were pooled in the health sector since 2005 for improvement in infrastructure, availability of manpower, drugs etc at health facilities, thus increasing the costs of service provision at the public health facilities.

### Policy and research implications of study findings

Our estimates on cost of rural PHCs and CHCs could be used to undertake further analysis. These could help in doing cost-effectiveness analysis of various primary health services delivered at these levels. Further, these estimates could be utilized by government for setting up of similar level of health facilities in urban areas under National Health Mission in India. Our study findings can also be used to generate national health accounts and state health accounts which are currently being undertaken by the Ministry of Health in India.

Also, in recent years a number of publicly financed health insurance schemes have significantly transformed the health financing landscape of India. The number of people under cover of any health insurance scheme has increased from 75 million in 2007 to 302 million in 2010 which is approximately one-fourth of the total population of India [22]. Around 46% of the health insurance spending comes from government /social health insurance schemes [22]. The purpose behind the public sponsored health schemes is to provide health equity, affordable health services to all the citizens. For the same, a scheme, *Rashtriya Swasthya Bima Yojana (RSBY*) provides a health insurance cover of 30,000 Indian rupees for a family of five living below poverty line [23]. In terms of provisioning, some studies have empanelled public sector health facilities such as CHCs who are reimbursed for the care provided. In Kerala, all the hospitals at the level of CHC and above are enrolled under RSBY-CHIs scheme [24]. The charges for various medical procedures, surgeries etc are predetermined by expert opinions based on market prices in view of lack of relevant literature [24]. Therefore, findings of our study could be used to revise the existing estimates and pay providers under these schemes.

### Limitations

We would like to highlight certain limitations of our analysis. Firstly, the numbers of primary health facilities taken in our study were not equal in two states of Haryana and Punjab. In case of Punjab, a large number of PHCs were upgraded as Block PHCs which had infrastructure resembling more to that of a CHC. Similarly, a number of new health facilities–Mini PHCs were created which were managed by the Local Governments. Hence, the numbers of PHCs which resemble the actual norms of a typical PHC were relatively less, resulting in a smaller sampling frame, ultimately resulting in relatively lesser number of overall PHCs drawn from the state. Secondly, we did not undertake a time-motion study to assess time contribution of staff performing multiple tasks. While we do acknowledge presence of more robust time-motion studies to understand the time allocation patterns [25], however, activity patterns at primary and community health centres preclude the application of such methods. Moreover, omission of a detailed time-motion study, and application of methods used in our study have also been justified in other studies [11, 21, 26]. Thirdly, it is important to highlight that for a number of services, resources were available at pooled facility level only. We used standard apportioning techniques which are recommended elsewhere [25]. Health facility and public health MIS systems should provide disaggregated data to help determine specific specialty costs in a more robust manner in future studies. Fourthly, our estimates of cost are reflective of the current level of infrastructure and services delivered. However, these may not be completely representative of costs in an ideal scenario, as envisaged by IPHS [2, 3]. For example the overall number of human resources and the mix of staff were not exactly as per recommendations. Similarly, these hospitals may not have had the desired set of all medicines available throughout the year. In a study done during similar period it was reported that the 47.8% of the basket of medicines are available in public sector district hospitals in Punjab [27]. Similarly, in another study from Punjab state it was pointed out that there is shortage of medicines which leads to high out-of-pocket expenditures for patients [28]. Fifth, we would also like to highlight that our estimates on cost of care do not account for the out-of-pocket expenditures which people incur in public sector hospitals. However, there is abundant evidence available on the extent of OOP in public sector hospitals of North India [29–33].

We also acknowledge that the health facilities varies a lot in terms of resources available, types of services provided, utilization and coverage of services among different states in India. This suggests that our results may not be generalized for national level. A larger sample of health facilities from different states of India would provide more representative results. We also found significant differences in the unit cost for providing same services at different facilities of same level. The difference may be due to difference in the number of beneficiaries who have availed that particular service in different facilities. The latter influenced the unit cost of service provision.

In our analysis, besides presenting facility level estimates of cost, we used the bootstrap method to generate the population mean for unit cost and its dispersion. Bootstrap method was used as the original sample was too less to use a parametric method for generating estimates of central tendency and dispersion which could be used to represent the population level health system costs. We acknowledge that the bootstrap method is only useful if the original sample follows more or less the same distribution as the original population. In order to be certain this is the case the sample size needs to be large enough. But what is large enough, is an unresolved question in statistical literature.

The problem in determining the appropriate sample size is the same when using the central limit theorem to determine the population mean. A large enough sample size can ensure that the population of sample means is normally distributed around the population mean. But again, what is large enough? While Good suggest a minimum sample size of 50, Yung and Chan (1999) review the evidence on use of bootstrapping with small samples, and conclude that it is not possible to give a simple recommendation for the minimum sample size for bootstrap method [34–38]. In general bootstrap method has been reported to compare favorably over asymptotic methods, even with small original sample sizes. In case of our analysis, the health facilities were sampled randomly. Also, the sampled facilities resemble the population of health facilities, i.e. PHCs and CHCs in those states in terms of manpower strength, availability of medicines, capital infrastructure etc. Hence we believe that the sampled facilities are a random representation of the population mean, and as a result application of bootstrap is justified.

## Conclusion

Overall, although the costing studies have been done on primary health care before, to our knowledge, there has been no study at all on the costing of community health centers in India. Further, our study provides cost estimates of health service delivery at primary and community health centers separately. The evidence provided in our study can be used as a basis for setting up of urban PHCs and CHCs under the National Health Mission and for evaluation of inclusion of services & cost levied on them under the Universal Health coverage. Since the government is providing most of the health services free of cost, the results can be used to see the extent to which subsidies have been cost effective to the government. The estimates can be used by agencies to revise the annual premium involved in community health insurance schemes. More such study needs to be carried out on a bigger scale to get better idea of the public health expenditure.

## Supporting Information

S1 Data Data. (XLSX)

S1 Fig Tornado diagram for sensitivity analysis of input costs at Primary Health Centers. (DOCX)

S2 Fig Tornado diagram for sensitivity analysis of input costs at Community Health Centers. (DOCX)

S1 Table Health care services being provided in seven Primary Health Centers of north India. (DOCX)

S2 Table Health care services being provided in seven Community Health Centers of north India. (DOCX)

S3 Table Costing assumptions and apportioning statistics. (DOCX)

S1 Tool Cost data collection tool for Primary Health Centre. (DOCX)

S2 Tool Cost data collection tool for Community Health Centre. (DOCX)
