# Supplementary material for: Cost of Delivering Health Care Services in Public Sector Primary and Community Health Centres in North India
Source: PLoS One. 2016 Aug 18;11(8):e0160986. doi: 10.1371/journal.pone.0160986 (PMC4990301; doi:10.1371/journal.pone.0160986)
Supplement: S2 Fig — (DOCX) [file pone.0160986.s003.docx]

S2 Figure: Tornado diagram for sensitivity analysis of input costs at Community Health Centers
