# Supplementary material for: Cost of Delivering Health Care Services in Public Sector Primary and Community Health Centres in North India
Source: PLoS One. 2016 Aug 18;11(8):e0160986. doi: 10.1371/journal.pone.0160986 (PMC4990301; doi:10.1371/journal.pone.0160986)
Supplement: S1 Table — (DOCX) [file pone.0160986.s004.docx]

S1 Table: Health care services being provided in seven Primary Health Centers of north India.

| **Primary health centers (PHCs) (n=7)** | | | | | | | |
| --- | --- | --- | --- | --- | --- | --- | --- |
| **PHC Id No.** | **P1** | **P2** | **P3** | **P4** | **P5** | **P6** | **P7** |
| Daily General Medical OPD | Y | Y | Y | Y | Y | Y | Y |
| Daily IPD services | Y | Y | Y | Y | Y | Y | Y |
| Minor surgery for management of abscess, wound, fractures, snake bites etc | Y | Y | Y | Y | Y | N | N |
| Maternal and child health |  |  |  |  |  |  |  |
| a. Ante-natal care | Y | Y | Y | Y | Y | Y | Y |
| b. Intra natal care (24 - hour delivery services both normal and assisted | Y | Y | Y | Y | Y | Y | Y |
| c. Post-natal care | Y | Y | Y | Y | Y | Y | Y |
| d. New born Care | Y | Y | Y | Y | Y | Y | Y |
| e. Child care including immunization | Y | Y | Y | Y | Y | Y | Y |
| f. Family Planning | Y | Y | Y | Y | Y | Y | Y |
| g. MTP | Y | Y | Y | Y | Y | Y | Y |
| Management of RTI/STI | Y | Y | Y | Y | Y | Y | Y |
| Other Services |  |  |  |  |  |  |  |
| Nutrition services. | Y | Y | Y | Y | Y | N | N |
| School Health programmes. | Y | Y | Y | Y | Y | Y | Y |
| Promotion of safe water supply and basic sanitation | Y | Y | Y | Y | Y | Y | Y |
| Prevention and control of locally endemic diseases | Y | Y | Y | Y | Y | Y | Y |
| Disease surveillance and control of epidemics. | Y | Y | Y | Y | Y | Y | Y |
| Collection and reporting of vital statistics. | Y | Y | Y | Y | Y | Y | Y |
| Education about health/behavior change communication | Y | Y | Y | Y | Y | Y | Y |
| National Health Programmes including HIV/AIDS control programme | Y | Y | Y | Y | Y | Y | Y |
| AYUSH services as per local preference. | Y | Y | Y | Y | Y | N | N |
| Rehabilitation services | Y | Y | Y | Y | Y | Y | Y |
| Physical Infrastructure |  |  |  |  |  |  |  |
| OPD rooms/cubicles | Y | Y | Y | Y | Y | Y | Y |
| Family Welfare Clinic | Y | Y | Y | Y | Y | Y | Y |
| Waiting room for patients | Y | Y | Y | Y | Y | N | N |
| Emergency Room/Casualty | Y | Y | Y | N | Y | N | Y |
| Separate wards for males and females | Y | Y | Y | N | Y | Y | Y |
| labour room | Y | Y | Y | Y | Y | Y | Y |
| Laboratory | Y | Y | Y | Y | Y | Y | Y |
| Immunization facilities | Y | Y | Y | Y | Y | Y | Y |
| vehicle service | Y | Y | Y | Y | Y | Y | Y |
| **Specialty Diagnostic Services/Tests** | | | | | | | |
| **I. URINE EXAMINATION** |  |  |  |  |  |  |  |
| i. Specific gravity and PH. | Y | Y | Y | Y | Y | Y | Y |
| ii. Test for glucose. | Y | Y | Y | Y | Y | Y | Y |
| iii. Test for protein (albumen). | Y | Y | Y | Y | Y | Y | Y |
| iv. Test for bile pigments and bile salts. | Y | Y | Y | Y | Y | N | N |
| v. Test for ketone bodies. | Y | Y | N | Y | Y | N | N |
| vi. Microscopic examination. | Y | Y | Y | Y | Y | Y | Y |
| **II STOOL EXAMINATION** |  |  |  |  |  |  |  |
| i. Gross examination. | Y | Y | Y | Y | Y | Y | Y |
| ii. Microscopic examination | Y | Y | Y | Y | Y | Y | Y |
| **III BLOOD EXAMINATION** |  |  |  |  |  |  |  |
| i. Collection of blood specimen by finger prick technique | Y | Y | Y | Y | Y | Y | Y |
| ii. Hemoglobin estimation. | Y | Y | Y | Y | Y | Y | Y |
| iii. RBC count. | Y | Y | Y | Y | Y | Y | Y |
| iv. WBC count (total and differential). | Y | Y | Y | Y | Y | Y | Y |
| v. Preparation, staining and examination of thick and thin blood smears for malaria parasites and for microfilaria. | Y | Y | Y | Y | Y | Y | Y |
| vi. Erythrocyte Sedimentation Rate. | Y | Y | Y | Y | Y | Y | Y |
| vii. VDRL. | Y | Y | Y | Y | Y | Y | Y |
| viii. Blood grouping and Rh typing. | Y | Y | Y | Y | Y | Y | Y |
| ix. Rapid HIV and STI Screening test | Y | Y | N | N | Y | N | N |
| **IV. SPUTUM EXAMINATION** |  |  |  |  |  |  |  |
| Mycobacterium tuberculosis (if PHC is recognized as microscopy centre under RNTCP) | Y | Y | Y | Y | Y | Y | Y |
| **V.SEMEN EXAMINATION** |  |  |  |  |  |  |  |
| i. Microscopic examination. | N | N | N | N | N | N | N |
| ii. Sperm count motility, morphology etc. | N | N | N | N | N | N | N |
| **VI THROAT SWAB PREPARATION** |  |  |  |  |  |  |  |
| i. Collection of throat swab and examination for diphtheria | Y | Y | Y | Y | Y | N | N |
| **VII.TEST SAMPLES FOR DRINKING WATER** |  |  |  |  |  |  |  |
| i. Testing of samples for gross impurities. | Y | Y | Y | Y | Y | N | N |
| ii. Rapid tests for detecting fecal contamination by HYS strip test. | Y | Y | Y | Y | Y | N | N |
| iii. Residual chlorine in drinking water by testing kits | Y | Y | Y | Y | Y | Y | Y |
