# Supplementary material for: Cost of Delivering Health Care Services in Public Sector Primary and Community Health Centres in North India
Source: PLoS One. 2016 Aug 18;11(8):e0160986. doi: 10.1371/journal.pone.0160986 (PMC4990301; doi:10.1371/journal.pone.0160986)
Supplement: S2 Table — (DOCX) [file pone.0160986.s005.docx]

S2 Table: Health care services being provided in seven Community Health Centers of north India.

| Community health centers (CHCs) (n=7) | | | | | | | |
| --- | --- | --- | --- | --- | --- | --- | --- |
| CHCs Identification number | C1 | C2 | C3 | C4 | C5 | C6 | C7 |
| **Services** | | | | | | | |
| **OPD Services** | | | | | | | |
| General Medicine | Y | Y | Y | Y | Y | Y | Y |
| General Surgery | Y | Y | Y | N | Y | Y | N |
| Obstetrics &Gynecology | Y | Y | Y | Y | Y | Y | N |
| Pediatrics | Y | Y | Y | Y | Y | Y | N |
| Dental | Y | Y | Y | Y | Y | Y | Y |
| AYUSH | Y | Y | Y | Y | Y | Y | N |
| **IPD Services** | | | | | | | |
| General Medicine | Y | Y | Y | Y | Y | Y | Y |
| General Surgery | Y | Y | Y | Y | Y | Y | N |
| Obstetrics &Gynecology | Y | Y | Y | Y | Y | Y | N |
| Pediatrics | Y | Y | Y | Y | Y | Y | N |
| Dental | Y | Y | Y | Y | Y | Y | N |
| AYUSH | Y | Y | Y | Y | Y | Y | N |
| **Other Services** | | | | | | | |
| Indoor Treatment/Wards | Y | Y | Y | Y | Y | Y | Y |
| 24 hrs Emergency and Medico-legal | Y | Y | Y | Y | Y | Y | Y |
| MCH Services (including high risk pregnancies) & Newborn stabilization unit | Y | Y | Y | Y | Y | Y | Y |
| Pharmacy | Y | Y | Y | Y | Y | Y | Y |
| Dentistry | Y | Y | Y | Y | Y | Y | Y |
| Laboratory | Y | Y | Y | Y | Y | Y | Y |
| Family Planning Services | Y | Y | Y | Y | Y | Y | Y |
| Immunization | Y | Y | Y | Y | Y | Y | Y |
| Blood storage | Y | Y | Y | Y | Y | Y | N |
| Operation Theatre | Y | Y | Y | Y | Y | Y | N |
| ECG | Y | Y | Y | Y | Y | Y | Y |
| 24 Hrs Ambulance | Y | Y | Y | Y | Y | Y | Y |
| **Specialty Diagnostic Services/Tests** | | | | | | | |
| **I CLINICAL PATHOLOGY** |  |  |  |  |  |  |  |
| **a) Hematology** |  |  |  |  |  |  |  |
| Hemoglobin estimation | Y | Y | Y | Y | Y | Y | Y |
| Total Leucocytes count | Y | Y | Y | Y | Y | Y | Y |
| Differential Leucocyte count | Y | Y | Y | Y | Y | Y | Y |
| Absolute Eosinophil count | Y | Y | Y | Y | Y | Y | Y |
| Reticulocyte count | Y | Y | Y | Y | Y | Y | Y |
| Total RBC count | Y | Y | Y | Y | Y | Y | Y |
| E.S.R. | Y | Y | Y | Y | Y | Y | Y |
| Peripheral Blood Smear | Y | Y | Y | Y | Y | Y | Y |
| Malaria/Filaria Parasite | Y | Y | Y | Y | Y | Y | Y |
| Platelet count | Y | Y | Y | Y | Y | Y | Y |
| Packed Cell volume | Y | Y | Y | Y | Y | Y | Y |
| Blood grouping | Y | Y | Y | Y | Y | Y | Y |
| Rh typing | Y | Y | Y | Y | Y | Y | Y |
| Blood Cross matching | Y | Y | Y | Y | Y | Y | Y |
| **b) Urine Analysis** |  |  |  |  |  |  |  |
| Urine for Albumin, Sugar, Deposits, bile salts, bile pigments, acetone, specific gravity, Reaction (pH) | Y | Y | Y | Y | Y | Y | Y |
| **c) Stool Analysis Stool for Ovacyst (Eh)** |  |  |  |  |  |  | Y |
| Hanging drop for V. Cholera | Y | Y | Y | Y | Y | Y | N |
| Occult blood | Y | Y | Y | Y | Y | Y | N |
| **II PATHOLOGY** |  |  |  |  |  |  |  |
| a) Sputum | Y | Y | Y | Y | Y | Y | Y |
| Sputum cytology | Y | Y | Y | Y | Y | Y | N |
| **III MICROBIOLOGY** |  |  |  |  |  |  |  |
| Smear for AFB, KLB | Y | Y | Y | Y | Y | Y | Y |
| Grams Stain for Throat swab, sputum etc. | Y | Y | Y | Y | Y | Y | Y |
| **IV SEROLOGY** |  |  |  |  |  |  |  |
| VDRL | Y | Y | Y | Y | Y | Y | Y |
| Pregnancy test (Urine gravidex) | Y | Y | Y | Y | Y | Y | Y |
| WIDAL test | Y | Y | Y | Y | Y | Y | Y |
| **V BIOCHEMISTRY** |  |  |  |  |  |  |  |
| Blood Sugar | Y | Y | Y | Y | Y | Y | Y |
| Blood urea | Y | Y | Y | Y | Y | Y |  |
| Liver function tests | Y | Y | Y | Y | Y | Y | Y |
| Kidney function tests | Y | Y | Y | Y | Y | Y |  |
| Blood lipid profile | Y | Y | Y | Y | Y | Y | Y |
| **VI CARDIAC INVESTIGATIONS** |  |  |  |  |  |  |  |
| a) ECG | Y | Y | Y | Y | Y | Y | Y |
| **VII OPHTHALMOLOGY** |  |  |  |  |  |  |  |
| Refraction by using Snellen's chart | Y | Y | Y | Y | Y | Y | Y |
| Retinoscopy | N | N | N | N | N | N | N |
| Ophthalmoscope | Y | Y | N | Y | Y | Y | N |
| **VIII RADIOLOGY** | | | | | | | |
| X ray for Chest, Skull, Spine, Abdomen, bones | Y | Y | Y | Y | Y | Y | Y |
| Dental X ray | Y | Y | Y | Y | Y | Y | Y |
| Ultrasonography (Desirable) | Y | Y | Y | Y | Y | Y | Y |
