# Supplementary material for: Cost of Delivering Health Care Services in Public Sector Primary and Community Health Centres in North India
Source: PLoS One. 2016 Aug 18;11(8):e0160986. doi: 10.1371/journal.pone.0160986 (PMC4990301; doi:10.1371/journal.pone.0160986)
Supplement: S3 Table — (DOCX) [file pone.0160986.s006.docx]

S3 Table: Costing assumptions and apportioning statistics

| Costing head | Sources of Information | Basic Analysis | Apportioning statistics for Joint costs |
| --- | --- | --- | --- |
| Salaries of human resource | Pay slips of Staff and account records from District health office | Annual salaries of the health staff of the health facility were calculated including the TA/DAs, cost of additional perks provided. | As per the proportion of time spent by an individual on various activities or services |
| Building (Space and Rent) | Space : Facility survey by the investigators  Rent: by interviewing key informants for market rental price for 100 sq feet of space | The annual rental value of the space was calculated by obtaining the market rental values of the place. | As per the proportion of time spent on different activities which were conducted in the particular space. |
| Furniture and Equipments  (Quantity, price and average life) | Quantity: Stock registers and facility survey by investigators  Price: Rate contract of state governments, market price by physical interviews with distributers, dealers and relevant websites.  Average life: literature review, interviews with staff at health facility | The onetime costs of purchase of furniture and equipments were annualized for their average life. | The annualized cost was then apportioned as per the proportion of time the enlisted items were utilized in various activities. |
| Drugs (Price and Quantity) | Price: Rate contract of state government,  Book on drug prices “IDR triplei Compendium [2011 Issue 5]  Market price lists were also obtained from the local distributors.  Quantity: stock registers in the health facilities were referred. | The annual amount spent on drugs was calculated by multiplying quantity procured within financial year and unit price for each drug. | As per proportion of the beneficiaries utilizing these drugs under various healthcare services being provided at the facility. |
| Consumables (Price and Quantity) | Quantity: To record the annual utilization within last year, the stock Registers were checked.  Prices: Rate contract of state governments,  Market price from the distributors | The annual expenditure on consumables was calculated from the quantity used and unit prices | As per proportion of beneficiaries utilizing drugs under various healthcare services. |
