# Supplementary material for: Cost of Delivering Health Care Services in Public Sector Primary and Community Health Centres in North India
Source: PLoS One. 2016 Aug 18;11(8):e0160986. doi: 10.1371/journal.pone.0160986 (PMC4990301; doi:10.1371/journal.pone.0160986)
Supplement: S1 Tool — (DOCX) [file pone.0160986.s007.docx]

**Cost data collection tool**

*Facility type: Primary Health Centre (PHC)*

Information about the facility

| Interview Date | __/__/__ |
| --- | --- |
| State name |  |
| District Name |  |
| Facility Type |  |
| Facility Name |  |
| Investigator Name |  |

This tool intends to collect information pertaining to following heads for the last ***financial year 2012-13***

| **S.NO** | Different heads for cost data collection | Put tick mark at the end of interview |
| --- | --- | --- |
| **1** | General information |  |
| **2 &3** | Personnel |  |
| **4** | Record on services delivered/Vaccination details |  |
| **5** | Sources of revenue |  |
| **6** | Details regarding population covered |  |
| **7** | Equipment |  |
| **8** | Consumable drugs |  |
| **9** | Consumable Materials and Supplies/ Details on any other Kit/ Supplies as part of health programmes |  |
| **10 & 11** | Physical infrastructure |  |
| **12** | Non-Medical items |  |
| **13** | IEC material |  |
| **14** | Stationary |  |
| **15** | Utility / Overheads |  |
| **16** | Laboratory test |  |
| **17** | Referral transport |  |
| **18** | Incentives paid |  |
| **19** | Grants utilised/ Amount spent under different schemes |  |
| **20** | Morbidity profile of the patient |  |
| **21** | Time allocation sheet |  |

***Section 1 General Information***

***Table 1: Interview with the head of the facility or person In-charge***

1. Is it a 24x7 PHC? ___________ (Yes/No)
2. Please tell me how many days per week this facility is closed? : __________ (Days per week)
3. Please tell me how many hours per day this facility is open (If B. = No)? : _________ (Hours per day)
4. If the facility remains closed on Public holidays then mention total public holidays in last year: _________(Days in year)
5. Average length of stay of IPD patients of the facility:

Mention the number of days of stay of 50 patients of facility of financial year 2012-13

|  |  |  |  |  |  |  |  |  |  |
| --- | --- | --- | --- | --- | --- | --- | --- | --- | --- |
|  |  |  |  |  |  |  |  |  |  |
|  |  |  |  |  |  |  |  |  |  |
|  |  |  |  |  |  |  |  |  |  |
|  |  |  |  |  |  |  |  |  |  |
|  |  |  |  |  |  |  |  |  |  |
|  |  |  |  |  |  |  |  |  |  |
|  |  |  |  |  |  |  |  |  |  |
|  |  |  |  |  |  |  |  |  |  |
|  |  |  |  |  |  |  |  |  |  |
|  |  |  |  |  |  |  |  |  |  |
|  |  |  |  |  |  |  |  |  |  |

**Section 2 & 3: Human resource-Salary and fringe benefits details**

*Table 2: Salary details: Details for each person separately using codes giv*en below

| ***Staff No. Code**** | ***Job title^@^*** | ***Speciality*** | ***Services (OPD=1, IPD=2, Out-reach=3,OPD+IPD=4, All= 5, OPD+OR=6, IPD+ OR=7)*** | ***Monthly gross salary***  ***(inclusive of all allowances or deductions)*** | ***Annual Incentive received for trainings***  ***(TA/DA received for trainings)*** | ***Period/days of posting in the year 2012-13^$^*** | ***Days of absence from this health facility in the period of posting in the year 2012-13^$$^*** |
| --- | --- | --- | --- | --- | --- | --- | --- |
|  |  |  |  |  |  |  |  |
|  |  |  |  |  |  |  |  |
|  |  |  |  |  |  |  |  |
|  |  |  |  |  |  |  |  |
|  |  |  |  |  |  |  |  |

*Medical Officer- MBBS = 1, MO –AYUSH= 2, Dental officer=3, Accountant/Clerk=4, Pharmacist=5, Pharmacist AYUSH=6, Nurse-midwife (Staff-Nurse)=7, Health workers (F)=8, Health worker (M) =9. Health Asstt. (Male) = 10, Health Asstt. (Female)/LHV=11, Health Educator= 12, Data entry cum computer operator=13, Laboratory Technician=14, Cold Chain & Vaccine Logistic Assistant=15, Multi-skilled Group D worker=16, Sanitary worker cum watchman=17, Sanitary worker=18, Watchman=19. *For more than one person of a particular category, use alphabetic prefixes. For e.g. if there are 2 medical officers use code 1a and 1b.*

*^@^Add extra rows, if more perssonel*

*^$^Include any person posted during 2012-13, but now transferred/not posted and include any person not posted at this facility during 2012-13, but providing services in PHC for few days or week/ month/ year.*

*^$$^Leave of any nature, training days, etc.*

***Table 2: Salary details: Details for each person separately using codes giv*en below**

| ***Staff No. Code*** | ***Job title*** | ***Speciality*** | ***Services (OPD=1, IPD=2, Out-reach=3,OPD+IPD=4, All= 5, OPD+OR=6, IPD+ OR=7)*** | ***Monthly gross salary***  ***(inclusive of all allowances or deductions)*** | ***Annual Incentive received for trainings***  ***(TA/DA received for trainings)*** | ***Period/days of posting in the year 2012-13*** | ***Days of absence from this health facility in the period of posting in the year 2012-13*** |
| --- | --- | --- | --- | --- | --- | --- | --- |
|  |  |  |  |  |  |  |  |
|  |  |  |  |  |  |  |  |
|  |  |  |  |  |  |  |  |
|  |  |  |  |  |  |  |  |
|  |  |  |  |  |  |  |  |
|  |  |  |  |  |  |  |  |
|  |  |  |  |  |  |  |  |
|  |  |  |  |  |  |  |  |
|  |  |  |  |  |  |  |  |
|  |  |  |  |  |  |  |  |
|  |  |  |  |  |  |  |  |

Medical Officer- MBBS = 1, MO –AYUSH= 2, Dental officer=3, Accountant/Clerk=4, Pharmacist=5, Pharmacist AYUSH=6, Nurse-midwife (Staff-Nurse)=7, Health workers (F)=8, Health worker (M) =9. Health Asstt. (Male) = 10, Health Asstt. (Female)/LHV=11, Health Educator= 12, Data entry cum computer operator=13, Laboratory Technician=14, Cold Chain & Vaccine Logistic Assistant=15, Multi-skilled Group D worker=16, Sanitary worker cum watchman=17, Sanitary worker=18, Watchman=19.

***Table 2: Salary details: Details for each person separately using codes giv*en below**

| ***Staff No. Code*** | ***Job title*** | ***Speciality*** | ***Services (OPD=1, IPD=2, Out-reach=3,OPD+IPD=4, All= 5, OPD+OR=6, IPD+ OR=7)*** | ***Monthly gross salary***  ***(inclusive of all allowances or deductions)*** | ***Annual Incentive received for trainings***  ***(TA/DA received for trainings)*** | ***Period/days of posting in the year 2012-13*** | ***Days of absence from this health facility in the period of posting in the year 2012-13*** |
| --- | --- | --- | --- | --- | --- | --- | --- |
|  |  |  |  |  |  |  |  |
|  |  |  |  |  |  |  |  |
|  |  |  |  |  |  |  |  |
|  |  |  |  |  |  |  |  |
|  |  |  |  |  |  |  |  |
|  |  |  |  |  |  |  |  |
|  |  |  |  |  |  |  |  |
|  |  |  |  |  |  |  |  |
|  |  |  |  |  |  |  |  |
|  |  |  |  |  |  |  |  |
|  |  |  |  |  |  |  |  |

Medical Officer- MBBS = 1, MO –AYUSH= 2, Dental officer=3, Accountant/Clerk=4, Pharmacist=5, Pharmacist AYUSH=6, Nurse-midwife (Staff-Nurse)=7, Health workers (F)=8, Health worker (M) =9. Health Asstt. (Male) = 10, Health Asstt. (Female)/LHV=11, Health Educator= 12, Data entry cum computer operator=13, Laboratory Technician=14, Cold Chain & Vaccine Logistic Assistant=15, Multi-skilled Group D worker=16, Sanitary worker cum watchman=17, Sanitary worker=18, Watchman=19.

***Table 3: Details of annual allowances received (Interviews and record review)***

| ***Staff No. Code*** | ***Government residence*** | | | ***Transport facility*** | | ***Uniform provided/ allowance*** | | | *Any other allowance* |
| --- | --- | --- | --- | --- | --- | --- | --- | --- | --- |
|  | *Square meter or square feet of the house building or rooms provided*  *(Do mention the unit of data collection)* | *Square meter or square feet of the open area in the accommodation provided* | *Amount paid in a year or How much would you pay if you would rent this house i.e. monthly rental price*12?* | *Amount paid in a year* | *Vehicle name and year of make, if provided free* | *Times per year*  *(a)* | *Unit cost of uniform*  *(b)* | *Amount incurred on uniform (a*b) or*  *If unit cost not available ask,*  *“For how much it will be available from market, if bought on its own?”* |  |
|  |  |  |  |  |  |  |  |  |  |
|  |  |  |  |  |  |  |  |  |  |
|  |  |  |  |  |  |  |  |  |  |
|  |  |  |  |  |  |  |  |  |  |
|  |  |  |  |  |  |  |  |  |  |
|  |  |  |  |  |  |  |  |  |  |
|  |  |  |  |  |  |  |  |  |  |
|  |  |  |  |  |  |  |  |  |  |
|  |  |  |  |  |  |  |  |  |  |
|  |  |  |  |  |  |  |  |  |  |
|  |  |  |  |  |  |  |  |  |  |
|  |  |  |  |  |  |  |  |  |  |
|  |  |  |  |  |  |  |  |  |  |
|  |  |  |  |  |  |  |  |  |  |
|  |  |  |  |  |  |  |  |  |  |
|  |  |  |  |  |  |  |  |  |  |

**Section 4: Annual services delivered** *(*Facility reports to be reviewed and not areas reports)*

*Table 4a: Annual services delivered (Record based) (If data is collected for less than 1 year than mention the period)*

| ***Codes*** | ***Services delivered*** |  | ***Actual services delivered in OPD during last year*** | ***Actual services delivered in IPD during last year*** |
| --- | --- | --- | --- | --- |
|  | *Ante natal care (HB estimation, BP, TT, Physical Exam, IFA)** | *Total ante natal check-ups visits at facility.*  *For e.g. 3 ante natal check-ups of a lady will be counter as 3 visits* |  | *NA* |
|  | *Institutional deliveries* | *Total in last year* | *NA* |  |
|  | *Post natal care ** | *Total post natal check-ups visits at facility* |  | *NA* |
|  | *New born care corner* | *Total new-borns admitted* | *NA* |  |
|  | *Immunisation (Total number of new children registered for immunization in year 2012-13Total under the facility)* | *Total at the facility* |  | *NA* |
|  | *Total number of old registered children forwarded for immunization in 2012-13.* | *Total at the facility* |  | *NA* |
|  | *Routine OPD (over 5)* | *Total at the facility*  *(Fever, Diarrhoea, Worm infestation, ARI, First aid and other consultations)* |  | *NA* |
|  | *Routine OPD (U-5 )* | *Total at the facility*  *Diarrhoea/ ARI/pneumonia/ fever/jaundice/malnutrition etc.* |  | *NA* |
|  | *Family Planning: Tubectomy* | *Motivation* |  | *NA* |
|  |  | *Procedure* | *NA* |  |
|  | *Family Planning: IUCD* | *Motivation* |  | *NA* |
|  |  | *Procedure* | *NA* |  |
|  | *Family planning: Counselling Oral pill and Condom distribution* | *Condom and Oral pill users of facility* |  | *NA* |
|  | *Special day care services*  *Primary management wounds*  *Primary management fracture*  *Primary management abscess drainage*  *Primary management burns* |  |  | *NA* |
|  | *AYUSH services* |  |  | *NA* |
|  | *Dental services* |  |  | *NA* |
|  | *IPD services* | *Malaria, Dengue, Typhoid, simple fever, dog and snake bites, poisonings, burn, pneumonia, dehydration, respiratory conditions, etc.* |  |  |
|  | *IPD services* | *Accidents, Trauma* |  |  |
|  | *Any other IPD service* |  |  |  |
|  | *DOTS provision* | *DOTS users at facility* |  | *NA* |
| *17.* | *Emergency ambulatory services* |  |  | *NA* |
| *18.* | *Laboratory tests done* |  |  |  |
| *19.* | *Food service/dietician* |  |  |  |
| *20.* | *Transport* |  |  |  |
| *21.* | *Security* |  |  |  |
| *22.* | *Sterilization* |  |  |  |
| *23.* | *Indoor Residual spray* |  |  |  |
| *24.* | *Registration* |  |  |  |
| *25.* | *OR (Outreach) activities* |  |  |  |
| *26.* | *School health program* |  |  |  |
| *27.* | *Adolescent health program (ARSH)* |  |  |  |
| *28.* | *Surveillance (IDSP)* |  |  |  |
| *29.* | *Other1* |  |  |  |
| *30.* | *Other2* |  |  |  |
| *31.* | *Other3* |  |  |  |

***

**Section 5: Sources of Revenue**

*Table 5: Sources of Revenue*

|  |  | **Amount collected during the period of data collection** |
| --- | --- | --- |
| 1 | Procedure fee (Medical dental combined) |  |
| 2 | Referral Charges |  |
| 3 | Medical certificate for driving license |  |
| 4 | Birth –death registration |  |
| 5 | Record checking for Birth –death |  |
| 6 | Issuing the card Birth –death |  |
| 7 | Any other (specify) |  |
|  | **Total user fee from 2012-13** |  |

**Section 6: Population covered under facility**

*Table 6: Details regarding population covered at the facility*

| Total population under the Public Health Centre | Total=  Male=  Female=  Children (under 5 years)=  Children (5-10 years)= |
| --- | --- |

**Section 7: Details of Equipments**

*Table 7a: Equipments and capital goods (Observation cum record review of stock registers)*

*(Equipments procured in year 2012-13 should not be captured, but condemn equipments in year 2012-13 should be captured)*

| ***Equipment*** | ***Quantity*** | ***Useful life of equipment*** | ***Utility (OPD=1, IPD=2, Out-reach=3,OPD+IPD=4, All= 5, OPD+OR=6, IPD+ OR=7)*** | ***List services for which it is used. Write serial number codes from section 21*** |
| --- | --- | --- | --- | --- |
| **Essential** |  |  |  |  |
| Normal Delivery Kit |  |  |  |  |
| Equipment for assisted vacuum delivery |  |  |  |  |
| Equipment for assisted forceps delivery |  |  |  |  |
| Standard Surgical Set (for minor procedures like episiotomies stitching). |  |  |  |  |
| Equipment for Manual Vacuum Aspiration |  |  |  |  |
| Equipment for New Born Care and Neonatal  Resuscitation. |  |  |  |  |
| IUCD insertion kit. |  |  |  |  |
| Equipment/reagents for essential laboratory  Investigations |  |  |  |  |
| Refrigerator |  |  |  |  |
| ILR (Small) and DF (Small) with Voltage Stabilizer |  |  |  |  |
| Cold Boxes (Small & Large): Small- one |  |  |  |  |
| Vaccine Carriers with 4 Icepacks: Two per SC  (maximum 2 per polio booth) + 1 for PHC. |  |  |  |  |
| Spare ice pack box: 8, 25 & 60 ice pack boxes per  vaccine carrier, Small cold box & Large cold box  Respectively. |  |  |  |  |
| Freeze Tag: 2 per ILR bimonthly |  |  |  |  |
| Thermometres |  |  |  |  |
| Ice box. |  |  |  |  |
| Binocular microscope |  |  |  |  |
| Monocular Microscope |  |  |  |  |
| Laproscope |  |  |  |  |
| Nebuliser |  |  |  |  |
| Stadio-meter |  |  |  |  |
| Coploscope |  |  |  |  |
| Cryotherapy equipment |  |  |  |  |
| Spirometer |  |  |  |  |
| Non-invasive ventilator |  |  |  |  |
| Dialysis machine |  |  |  |  |
| Haemoglobinometer |  |  |  |  |
| Semi-auto analyser |  |  |  |  |
| **Equipments for pap smear** |  |  |  |  |
| Cusco’s vaginal speculum (each of small, medium  and large size) |  |  |  |  |
| Sim’s vaginal speculum – single & double ended -  (each of small, medium and large size) |  |  |  |  |
| Anterior Vaginal wall retractor |  |  |  |  |
| Kidney tray |  |  |  |  |
| Bowl |  |  |  |  |
| Cheatle’s forceps |  |  |  |  |
| Proper light source/torch |  |  |  |  |
|  |  |  |  |  |
| **OT Equipment** |  |  |  |  |
| Boyles apparatus |  |  |  |  |
| EMO machine |  |  |  |  |
| Cardiac machine |  |  |  |  |
| Defibrillator for OT |  |  |  |  |
| E Ventilator for OT |  |  |  |  |
| Horizontal high pressure steriliser |  |  |  |  |
| Vertical high pressure steriliser 2/3drum |  |  |  |  |
| Shadow less lamp ceiling track mounted |  |  |  |  |
| Fumigation apparatus |  |  |  |  |
| Pulse oxymeter |  |  |  |  |
| Suction machine |  |  |  |  |
| **Others** |  |  |  |  |
| Needle cutter |  |  |  |  |
| Adult weighing machine |  |  |  |  |
| New born weighing machine |  |  |  |  |
|  |  |  |  |  |
|  |  |  |  |  |
|  |  |  |  |  |
|  |  |  |  |  |
|  |  |  |  |  |
|  |  |  |  |  |

*Table 7b: Special equipment for new born care corner*

|  | ***Quantity*** | ***Useful life of equipment*** | ***Utility (OPD=1, IPD=2, Out-reach=3,OPD+IPD=4, All= 5, OPD+OR=6, IPD+ OR=7)*** | ***List services for which it is used. Write serial number codes from section 21*** |
| --- | --- | --- | --- | --- |
| Open care system: radiant warmer, fixed height, with trolley, drawers, O2-bottles |  |  |  |  |
| Resuscitator (silicone resuscitation bag and mask with reservoir) hand-operated, neonate, 500ml |  |  |  |  |
| Weighing Scale, spring |  |  |  |  |
| Pump suction, foot operated |  |  |  |  |
| Thermometer, clinical, digital, 32-34 0C |  |  |  |  |
| Light examination, mobile, 220-12 V |  |  |  |  |
| Hub Cutter, syringe |  |  |  |  |
| Laryngoscope and Endotracheal intubation tubes  (neonatal). |  |  |  |  |
| Feeding tubes for baby. |  |  |  |  |

*Table 7c: Dental Equipments*

| ***Equipments*** | ***Quantity*** | ***Useful life of equipment*** | ***Utility (OPD=1, IPD=2, Out-reach=3,OPD+IPD=4, All= 5, OPD+OR=6, IPD+ OR=7)*** | ***List services for which it is used. Write serial number codes from section 21*** |
| --- | --- | --- | --- | --- |
| Amalgam carrier |  |  |  |  |
| Artery forceps |  |  |  |  |
| Autoclave |  |  |  |  |
| Bone cutter |  |  |  |  |
| Cheatel forceps |  |  |  |  |
| Dental chair |  |  |  |  |
| Dapper glass |  |  |  |  |
| Dressing drum (big) |  |  |  |  |
| Endo box |  |  |  |  |
| Electric BP apparatus |  |  |  |  |
| Glass mortar |  |  |  |  |
| Glass pestle |  |  |  |  |
| Glass bead sterilizer |  |  |  |  |
| Hammer chisel |  |  |  |  |
| Kidney tray |  |  |  |  |
| Matrix retainer (ivory) |  |  |  |  |
| Light cure machine |  |  |  |  |
| Mirror tops |  |  |  |  |
| Mirror handles |  |  |  |  |
| Plastic filling instruments |  |  |  |  |
| Probe |  |  |  |  |
| Tooth extraction forceps |  |  |  |  |
| Tweezers |  |  |  |  |
| Steel tray |  |  |  |  |
| Operating light |  |  |  |  |
| Halogen bulbs |  |  |  |  |
| High and low vacuum motored suction |  |  |  |  |
| Air rotor hand piece |  |  |  |  |
| Micro motor 35000 rpm |  |  |  |  |
| EMS scalar |  |  |  |  |
| 3 way syringe |  |  |  |  |
| X ray viewer |  |  |  |  |
| Compressor 3/4 HP with driver and filter |  |  |  |  |
| Stool with pneumatic cylinder |  |  |  |  |
| Surgical curette |  |  |  |  |
| Sterilizer |  |  |  |  |
| Dental X-ray tube head |  |  |  |  |
| Dental X-ray turner with stand |  |  |  |  |
| Development box |  |  |  |  |
| Needle holder |  |  |  |  |
| Electrical sterilizer |  |  |  |  |
| Cotton drum |  |  |  |  |
| Steel almirah |  |  |  |  |
| Needle destroyer |  |  |  |  |
| Revolving doctor chair |  |  |  |  |
| Visiting chair |  |  |  |  |
| Glass slab |  |  |  |  |
| Matrix bands (ivory) |  |  |  |  |
| Light cure composite |  |  |  |  |
| Mucus suction trap |  |  |  |  |
| Thermometer |  |  |  |  |

**Section 8: Details of drugs consumed in the facility (Take consumption data and not the supply data)**

*Table 8: Consumables. Drug (Review the stock- register and list the quantity of drugs in drug list sheets provided at the end of tool by using the sheets to photocopy index of drugs register and write quantity and utility against each drug as given below)*

| ***Name of drug*** | ***Quantity*** | ***Utility (OPD=1, IPD=2, Out-reach=3,OPD+IPD=4, All= 5, OPD+OR=6, IPD+ OR=7)*** | ***List services for which it is used. Write serial number codes from section 21*** |
| --- | --- | --- | --- |
| **Drugs for JSY/JSSK: Anti-natal Drugs** |  |  |  |
| Iron And Folic Acid - Dried Ferrous Sulphate Eq. To Ferrous Iron And Folic Acid |  |  |  |
| Methyledopa Anhydrous |  |  |  |
| Nifedipine |  |  |  |
| Nifedipine |  |  |  |
| Labetalol |  |  |  |
| Labetalol |  |  |  |
| Digoxin |  |  |  |
| Mag Sulphate |  |  |  |
| Folic Acid |  |  |  |
| **Drugs for JSY/JSSK: Intra-partum Drugs - Normal Delivery** |  |  |  |
| Ampicillin Trihydrate Eq. To Ampicilllin |  |  |  |
| Gentamycin Sulphate Eq. To Gentamycin |  |  |  |
| Ampicillin Trihydrate Eq. To Ampicillin Anhydrous |  |  |  |
| Amoxycilline Trihydrateeq.To Amoxycilline |  |  |  |
| Metronidazole |  |  |  |
| Nitrofurantoin |  |  |  |
| Doxycycline Hydrochloride |  |  |  |
| Methylegometrine Maleate |  |  |  |
| Misoprostole |  |  |  |
| Dicycloamine |  |  |  |
| Magnesium Sulphate Eq To Doxycycline |  |  |  |
| Oxytocin |  |  |  |
| Hyoscine Butyle Bromide |  |  |  |
| Hyoscine Butyle Bromide |  |  |  |
| Menadione Usp (Vit K3) |  |  |  |
| Sodium Lactate (Ringers Lactate) |  |  |  |
| Sodium Chloride |  |  |  |
| Dextrose Eq. To Dextrose Anhydrous |  |  |  |
| Sodium Bicarbonate |  |  |  |
| Sterile Water |  |  |  |
| Calcium Gluconate |  |  |  |
| Drotavarine |  |  |  |
| Povadine Iodine |  |  |  |
| Lignocane Hydrochloride |  |  |  |
| **Drugs for JSY/JSSK: Intrapartam Drugs - C section** |  |  |  |
| Metronidazole |  |  |  |
| Gentamycin Sulphate |  |  |  |
| Cefotaxime Sodium |  |  |  |
| Coxacillin |  |  |  |
| Oxytocin |  |  |  |
| Sensorcrain |  |  |  |
| Lignocaine Hydrochloride |  |  |  |
| Lignocaine Hydrochloride |  |  |  |
| Promethazene Hydrochloride |  |  |  |
| Declofenac |  |  |  |
| Sodium Lactate (Ringers Lactate) |  |  |  |
| Sodium Chloride |  |  |  |
| Dextrose Eq. To Dextrose Anhydrous |  |  |  |
| Sodium Bicarbonate |  |  |  |
| Menadione Usp (Vit K3) |  |  |  |
| Pentazocine Lactate |  |  |  |
| **Drugs for JSY/JSSK: Postnatal Drugs** |  |  |  |
| Iron And Folic Acid - Dried Ferrous Sulphate Eq. To Ferrous Iron And Folic Acid |  |  |  |
| Methyledopa Anhydrous |  |  |  |
| Nifedipine |  |  |  |
| Nifedipine |  |  |  |
| Labetalol |  |  |  |
| Labetalol |  |  |  |
| Digoxin |  |  |  |
| Mag Sulphate |  |  |  |
| Oxytocin |  |  |  |
| Hydroxyethyle Starch 6% Saline Solution |  |  |  |
| Paracetamol |  |  |  |
| Ibubrufen |  |  |  |
| Multivitamin |  |  |  |
| Domperidone |  |  |  |
| Anti D Immunoglobulin- Polyclonal Human Anti Rhd |  |  |  |
| Anti D Immunoglobulin- Polyclonal Human Anti Rhd |  |  |  |
| **Drugs for JSY/JSSK: Miscellaneous drugs** |  |  |  |
| Adrenaline Tartrate |  |  |  |
| Atropine Sulphate |  |  |  |
| Dopamine Hydrochloride |  |  |  |
| Bupivacane Hydrochloride |  |  |  |
| Betamethasone Phosphate |  |  |  |
| Halothane |  |  |  |
| Thiopentone |  |  |  |
| Veduronium Bromide |  |  |  |
| Ketamine Hydrochloride |  |  |  |
| Salbutamol Sulphate |  |  |  |
| Frusemide |  |  |  |
| Diazapam |  |  |  |
| Diazapam |  |  |  |
| Dexomethasone Sodium Phosphate Eq To Dexomethasone Phosphate |  |  |  |
| Etofyllin B Plus *, Anhydrous Theophylline** Combination |  |  |  |
| **Drugs for JSY/JSSK: Intra-natal consumables for normal delivery - DOSAGE FORM IS IN SIZE/QUANTITY** |  |  |  |
| Absorbent Cotton |  |  |  |
| Povidine Iodine Solution |  |  |  |
| Disposable Examination Gloves Latex |  |  |  |
| Surgical Gloves Sterile BIS |  |  |  |
| Hypodermic Syringe For Single Use BP/BIS |  |  |  |
| Hypodermic Needle For Single Use BP/BIS |  |  |  |
| Cotton Bandage |  |  |  |
| Absorbent Gauze |  |  |  |
| Surgical Spirit BP |  |  |  |
| Infusion Equipment BIS, IV Set With Hypodermic Needle |  |  |  |
| Intra Cath Cannulas For Single Use (IV Catheters) BIS |  |  |  |
| Chromic Catgut On Round Body Needle |  |  |  |
| Cord Lamp |  |  |  |
| Muccus Sucker |  |  |  |
| Medicated Soap |  |  |  |
| K-90, Plain Catheter |  |  |  |
| Floyes Catheter (Self Retaining Catheter) |  |  |  |
| **Drugs for JSY/JSSK: Intra-natal consumables for C section - size/quantity** |  |  |  |
| Absorbent Cotton |  |  |  |
| Povidine Iodine Solution |  |  |  |
| Sticking Plaster (Surgical Tape) |  |  |  |
| Hypodermic Syringe For Single Use BP/BIS |  |  |  |
| Hypodermic Needle For Single Use BP/BIS |  |  |  |
| Floyes Catheter (Self Retaining Catheter) |  |  |  |
| Infusion Equipment BIS, IV Set With Hypodermic Needle |  |  |  |
| Intra Cath Cannulas For Single Use (IV Catheters) BIS |  |  |  |
| Chromic Catgut On Round Body Needle |  |  |  |
| Cord Lamp |  |  |  |
| Section Tube |  |  |  |
| Spinal Needle Disposable Adiult BIS |  |  |  |
| Medicated Soap |  |  |  |
| K-90 Plain Catheter |  |  |  |
| Floyes Catheter (Self Retaining Catheter) |  |  |  |
| Urobag |  |  |  |
| Sponges |  |  |  |
| Cotton Bandage |  |  |  |
| Absorbent Gauze |  |  |  |
| Surgical Spirit BP |  |  |  |
| Mucus Sucker |  |  |  |
| Mersilk On Cutting Needle |  |  |  |
| Polyglycolic Acid, Braided, Coated And Absorbable, Half Circle Round Body |  |  |  |
| **Drugs for Drugs for JSY/JSSK: Newborn Care** |  |  |  |
| Adrenaline Tartrate |  |  |  |
| Amikacin |  |  |  |
| Aminophyline |  |  |  |
| Ampicillin Sodium Eq To Ampicillin Anhydrous |  |  |  |
| Calcium Gluconate |  |  |  |
| Dopamine |  |  |  |
| Gentamycin Sulphate |  |  |  |
| Dextrose Anhydrous |  |  |  |
| Phenobarbitone Sodium |  |  |  |
| Phenytoin Sodium Bp |  |  |  |
| Potassium Chloride |  |  |  |
| Sodium Bicarbonate |  |  |  |
| Sodium Chloride |  |  |  |
| Sterile Water |  |  |  |
| **STI/RTI kits** |  |  |  |
| **KIT NO.** |  |  |  |
| Kit 1 – Grey |  |  |  |
| Kit 2 – Green |  |  |  |
| Kit 3 – white |  |  |  |
| Kit 4 – Blue |  |  |  |
| Kit 5 – Red |  |  |  |
| Kit 6 – Yellow |  |  |  |
| **Drugs for TB** |  |  |  |
| Isoniazid |  |  |  |
| Isoniazid |  |  |  |
| Rifampicin |  |  |  |
| Pyrazinamide |  |  |  |
| Ethambutol |  |  |  |
| Streptomycin |  |  |  |
| Kanamycin |  |  |  |
| Amikacin |  |  |  |
| **Drugs for NCD: Cardiovascular** |  |  |  |
| Glyceryl Trinitrate |  |  |  |
| Isosorbide Dinitrate |  |  |  |
| Digoxin |  |  |  |
| Atenolol |  |  |  |
| Perindropil |  |  |  |
| Methyldopa |  |  |  |
| Propranolol Hydrochloride |  |  |  |
| Labetolol Hydrochloride |  |  |  |
| Metoprolol Tartrate |  |  |  |
| Nifedipine |  |  |  |
| Prazosin Hydrochloride |  |  |  |
| Enalpril |  |  |  |
| Telmisartan |  |  |  |
| Amlodipine |  |  |  |
| Digoxin |  |  |  |
| Simvastatin |  |  |  |
| **Drugs for NCD: Antidiabetics** |  |  |  |
| Glibenclamide |  |  |  |
| Glicazide |  |  |  |
| Insulin recombinant Neutral Human short acting |  |  |  |
| Insulin recombinant Neutral Human long acting |  |  |  |
| Metformin Hydrochloride |  |  |  |
| **Drugs for NCD: Diuretics** |  |  |  |
| Fursemide |  |  |  |
| Fursemide |  |  |  |
| Hydrochlorothiazide |  |  |  |
| Spironolactone |  |  |  |
| Medroxyprogesterone Acetate |  |  |  |
| **Drugs for NCD: Respiratory** |  |  |  |
| Aminophyline |  |  |  |
| Beclomethasone Dipropionate |  |  |  |
| Budesonide |  |  |  |
| Ipratropium Bromide |  |  |  |
| Salbutamol |  |  |  |
| Salbutamol |  |  |  |
| Salbutamol |  |  |  |
| Salbutamol |  |  |  |
| Theophyline |  |  |  |
| Ipratropium Bromide |  |  |  |
| Diphenhydramine Hydrochloride +Ammonium Chloride |  |  |  |
| Diphenhydramine Hydrochloride |  |  |  |
| Amonium Bicarb,Tinc |  |  |  |
| Bromhexine Hydrochloride |  |  |  |
| Bromhexine Hydrochloride |  |  |  |
|  |  |  |  |
|  |  |  |  |
|  |  |  |  |
|  |  |  |  |

**Section 9: Details of Consumables. Material and Supplies consumed in the facility**

*Table 9a: Consumables. Material and Supplies*

| ***Consumables*** | ***Quantity*** | ***Utility (OPD=1, IPD=2, Out-reach=3,OPD+IPD=4, All= 5, OPD+OR=6, IPD+ OR=7)*** | ***List services for which it is used. Write serial number codes from section 21*** |
| --- | --- | --- | --- |
| Bandages 4 meters* 5cm |  |  |  |
| Bandages 5 meters*10cm |  |  |  |
| Bandages 5 meters*15cm |  |  |  |
| Biowaste polythene |  |  |  |
| Blade 11 no. |  |  |  |
| Cotton wool absorbent surgical 500g packet |  |  |  |
| Crape bandage BP 3 mtr x 10cm |  |  |  |
| Crape bandage BP 3 mtr x 15cm |  |  |  |
| Crape bandage BP 3 mtr x 7.5cm |  |  |  |
| Disinfectant fluids (Phenly-Ltr) |  |  |  |
| Disposable surgical rubber gloves 6.5 |  |  |  |
| Gauge cloth 90cm*18 mtr |  |  |  |
| Infusion set vented with needle for single use (IV set) |  |  |  |
| IV Cannula 20 No. |  |  |  |
| IV cannula 24 no. |  |  |  |
| Mouth wash |  |  |  |
| Mucus suction trap |  |  |  |
| Spirit |  |  |  |
| Surgical tape 25mm*9.1 meter |  |  |  |
| Surgical tape 50mm*9.1 meter |  |  |  |
| Surgical tape 75mm*9.1 meter |  |  |  |
|  |  |  |  |
|  |  |  |  |
|  |  |  |  |
|  |  |  |  |

*Table 9b: Vaccine consumables*

|  | **Vaccine consumables** | **Vials used in 2012-13 for immunisation at the facility** |
| --- | --- | --- |
| 1 | BCG |  |
| 2 | DPT I+II+III+ Booster |  |
| 3 | Polio-O+ I+II+III+ Booster |  |
| 4 | Hepatitis B I+II+III |  |
| 5 | Pentavalent I+II+III |  |
| 5 | Measles |  |
| 6 | Vitamin A (Add the total doses) |  |
| 7 | TT |  |
| 8 | OPV booster |  |
| 9 | Rotavirus vaccine |  |
| 10 | JE dose 1 |  |
| 11 | Any other |  |
| 12 | Any other (Specify) |  |
|  |  |  |
|  |  |  |

*Table: 9c. Dental consumables*

| ***Dental consumables*** | ***Quantity*** | ***Utility (OPD=1, IPD=2, Out-reach=3,OPD+IPD=4, All= 5, OPD+OR=6, IPD+ OR=7)*** | ***List services for which it is used. Write serial number codes from section 21*** |
| --- | --- | --- | --- |
| Disposable syringe 2cc |  |  |  |
| Disposable syringe 5cc |  |  |  |
| Gloves 6.5, 7, 7.5 |  |  |  |
| Root canal Reamers (45-80) |  |  |  |
| Root canal Reamers (15-40) |  |  |  |
| Suture needles |  |  |  |
| Self- etching bond |  |  |  |
| Silver alloy |  |  |  |
| Sodium hypochlorite |  |  |  |
| Xylocaine |  |  |  |
| Kodak X-ray films |  |  |  |
| Developer and fixer |  |  |  |
| 8 spreaders (15-40) |  |  |  |
| Spreaders (45-80) |  |  |  |
| Kalsogen 10 |  |  |  |
| Formacresol |  |  |  |
| Orafil |  |  |  |
| Zinc phosphate cement |  |  |  |
| Zinc oxide |  |  |  |
| H-files |  |  |  |
| Cotton |  |  |  |
| Spirit |  |  |  |
| K files (15-40) |  |  |  |
| Kalgenol |  |  |  |
| Absorbent paper points (45-80) |  |  |  |
| Absorbent paper points (15-40) |  |  |  |
| Calcium with paste + CaOH2 powder |  |  |  |
| Diamond burs |  |  |  |
| Gutta percha points (15-40) |  |  |  |
| Gutta percha points (45-80) |  |  |  |
| GIC |  |  |  |
| Mercury |  |  |  |
| Polycarbonate cement |  |  |  |
| Pyrocresol (formacresol) |  |  |  |
| crepe bandage 8x10 cm |  |  |  |
| Cotton gauge |  |  |  |
| IV set |  |  |  |
| IV cannula no. 18 |  |  |  |
| IV cannula no. 20 |  |  |  |
| IV cannula no. 22 |  |  |  |
| IV cannula no. 24 |  |  |  |
| Mouth wash |  |  |  |
| Mask triple layer |  |  |  |
| Phenyl |  |  |  |
| Spirit |  |  |  |
| Savlon |  |  |  |
| Surgical tape 25mm*9.1 meter |  |  |  |
| Surgical tape 50mm*9.1 meter |  |  |  |
| Surgical tape 75mm*9.1 meter |  |  |  |
| Toilet acid |  |  |  |
|  |  |  |  |
|  |  |  |  |

**Section 10 and 11: Details of the Physical infrastructure**

*Table10: Physical infrastructure (Interview based)*

| ***Table 12 a: Particulars*** | ***Specify*** |
| --- | --- |
| Area of the building (Total area in Sq. ft.) (Covered space) |  |
| Area of the building (Total area in Sq. ft.) (Open space) |  |
| What is the rental price of 100 sq ft place where this centre is located? |  |
| Was there any expense on renovation or construction of accessory items during the period 2012-13 |  |

**Facility Check**

*Ask the head of the facility if you can make a tour of the facility to get some information on the building space, vehicles and equipment. Use the space below to draw a simple layout of the facility. Identify the type of service delivered in each room/space using the codes available. Specify a number for each room /space on the map.*

*Alternatively you can ask for map of the building with area measurements.*

***Thank the head of the facility and ask him if you can revisit the different rooms to complete measurements (****if required)* ***and make a closer observation.***

*Use the following table to fill in the required information for each room in the building(s)*

*You need to have a measuring instrument (used to calculate length and width of the room) with you to measure square meter surface area*

*You need to complete the following observations:*

*Record the measurements needed in* Table 12b

*Draw a sketch of the facility in the space available above*

*Complete* Table 13*with the inventory of furniture and items available in each room.*

***Facility space***

***N.B.***

_ ***Do not forget waiting areas*** *(some of them can be for adult only or children only).*

_ ***If any of the rooms are not used at the moment*** *indicate this in the service/ function column.*

**Sketch of the facility:**

*Table 11: Services delivered in different rooms in facility (Put 1 if particular service is delivered in a particular room)*

|  |  | ***Codes for Services delivered (Table 21)*** | | | | | | | | | | | | | | | | | | | | | | | | | | |
| --- | --- | --- | --- | --- | --- | --- | --- | --- | --- | --- | --- | --- | --- | --- | --- | --- | --- | --- | --- | --- | --- | --- | --- | --- | --- | --- | --- | --- |
| ***Name of room*** | ***Square meter***  ***Or***  ***feet*** | ***1*** | ***2*** | ***3*** | ***4*** | ***5*** | ***6*** | ***7*** | ***8*** | ***9*** | ***10*** | ***11*** | *12* | *13* | *14* | *15* | *16* | *17* | *18* | *19* | *20* | *21* | *22* | *23* | *24* | *25* | *26* | *27* |
|  |  |  |  |  |  |  |  |  |  |  |  |  |  |  |  |  |  |  |  |  |  |  |  |  |  |  |  |  |
|  |  |  |  |  |  |  |  |  |  |  |  |  |  |  |  |  |  |  |  |  |  |  |  |  |  |  |  |  |
|  |  |  |  |  |  |  |  |  |  |  |  |  |  |  |  |  |  |  |  |  |  |  |  |  |  |  |  |  |
|  |  |  |  |  |  |  |  |  |  |  |  |  |  |  |  |  |  |  |  |  |  |  |  |  |  |  |  |  |
|  |  |  |  |  |  |  |  |  |  |  |  |  |  |  |  |  |  |  |  |  |  |  |  |  |  |  |  |  |
|  |  |  |  |  |  |  |  |  |  |  |  |  |  |  |  |  |  |  |  |  |  |  |  |  |  |  |  |  |
|  |  |  |  |  |  |  |  |  |  |  |  |  |  |  |  |  |  |  |  |  |  |  |  |  |  |  |  |  |
|  |  |  |  |  |  |  |  |  |  |  |  |  |  |  |  |  |  |  |  |  |  |  |  |  |  |  |  |  |
|  |  |  |  |  |  |  |  |  |  |  |  |  |  |  |  |  |  |  |  |  |  |  |  |  |  |  |  |  |
|  |  |  |  |  |  |  |  |  |  |  |  |  |  |  |  |  |  |  |  |  |  |  |  |  |  |  |  |  |
|  |  |  |  |  |  |  |  |  |  |  |  |  |  |  |  |  |  |  |  |  |  |  |  |  |  |  |  |  |
|  |  |  |  |  |  |  |  |  |  |  |  |  |  |  |  |  |  |  |  |  |  |  |  |  |  |  |  |  |
|  |  |  |  |  |  |  |  |  |  |  |  |  |  |  |  |  |  |  |  |  |  |  |  |  |  |  |  |  |
|  |  |  |  |  |  |  |  |  |  |  |  |  |  |  |  |  |  |  |  |  |  |  |  |  |  |  |  |  |
|  |  |  |  |  |  |  |  |  |  |  |  |  |  |  |  |  |  |  |  |  |  |  |  |  |  |  |  |  |
|  |  |  |  |  |  |  |  |  |  |  |  |  |  |  |  |  |  |  |  |  |  |  |  |  |  |  |  |  |
|  |  |  |  |  |  |  |  |  |  |  |  |  |  |  |  |  |  |  |  |  |  |  |  |  |  |  |  |  |
|  |  |  |  |  |  |  |  |  |  |  |  |  |  |  |  |  |  |  |  |  |  |  |  |  |  |  |  |  |
|  |  |  |  |  |  |  |  |  |  |  |  |  |  |  |  |  |  |  |  |  |  |  |  |  |  |  |  |  |
|  |  |  |  |  |  |  |  |  |  |  |  |  |  |  |  |  |  |  |  |  |  |  |  |  |  |  |  |  |

|  |  | ***Codes for Services delivered (Table 21)*** | | | | | | | | | | | | | | | | | | | | | | | | | | |
| --- | --- | --- | --- | --- | --- | --- | --- | --- | --- | --- | --- | --- | --- | --- | --- | --- | --- | --- | --- | --- | --- | --- | --- | --- | --- | --- | --- | --- |
| ***Name of room*** | ***Square meter***  ***Or***  ***feet*** | ***1*** | ***2*** | ***3*** | ***4*** | ***5*** | ***6*** | ***7*** | ***8*** | ***9*** | ***10*** | ***11*** | *12* | *13* | *14* | *15* | *16* | *17* | *18* | *19* | *20* | *21* | *22* | *23* | *24* | *25* | *26* | *27* |
|  |  |  |  |  |  |  |  |  |  |  |  |  |  |  |  |  |  |  |  |  |  |  |  |  |  |  |  |  |
|  |  |  |  |  |  |  |  |  |  |  |  |  |  |  |  |  |  |  |  |  |  |  |  |  |  |  |  |  |
|  |  |  |  |  |  |  |  |  |  |  |  |  |  |  |  |  |  |  |  |  |  |  |  |  |  |  |  |  |
|  |  |  |  |  |  |  |  |  |  |  |  |  |  |  |  |  |  |  |  |  |  |  |  |  |  |  |  |  |
|  |  |  |  |  |  |  |  |  |  |  |  |  |  |  |  |  |  |  |  |  |  |  |  |  |  |  |  |  |
|  |  |  |  |  |  |  |  |  |  |  |  |  |  |  |  |  |  |  |  |  |  |  |  |  |  |  |  |  |
|  |  |  |  |  |  |  |  |  |  |  |  |  |  |  |  |  |  |  |  |  |  |  |  |  |  |  |  |  |
|  |  |  |  |  |  |  |  |  |  |  |  |  |  |  |  |  |  |  |  |  |  |  |  |  |  |  |  |  |
|  |  |  |  |  |  |  |  |  |  |  |  |  |  |  |  |  |  |  |  |  |  |  |  |  |  |  |  |  |
|  |  |  |  |  |  |  |  |  |  |  |  |  |  |  |  |  |  |  |  |  |  |  |  |  |  |  |  |  |
|  |  |  |  |  |  |  |  |  |  |  |  |  |  |  |  |  |  |  |  |  |  |  |  |  |  |  |  |  |
|  |  |  |  |  |  |  |  |  |  |  |  |  |  |  |  |  |  |  |  |  |  |  |  |  |  |  |  |  |
|  |  |  |  |  |  |  |  |  |  |  |  |  |  |  |  |  |  |  |  |  |  |  |  |  |  |  |  |  |
|  |  |  |  |  |  |  |  |  |  |  |  |  |  |  |  |  |  |  |  |  |  |  |  |  |  |  |  |  |
|  |  |  |  |  |  |  |  |  |  |  |  |  |  |  |  |  |  |  |  |  |  |  |  |  |  |  |  |  |
|  |  |  |  |  |  |  |  |  |  |  |  |  |  |  |  |  |  |  |  |  |  |  |  |  |  |  |  |  |
|  |  |  |  |  |  |  |  |  |  |  |  |  |  |  |  |  |  |  |  |  |  |  |  |  |  |  |  |  |
|  |  |  |  |  |  |  |  |  |  |  |  |  |  |  |  |  |  |  |  |  |  |  |  |  |  |  |  |  |
|  |  |  |  |  |  |  |  |  |  |  |  |  |  |  |  |  |  |  |  |  |  |  |  |  |  |  |  |  |
|  |  |  |  |  |  |  |  |  |  |  |  |  |  |  |  |  |  |  |  |  |  |  |  |  |  |  |  |  |

**Section 12: Details about non-medical items**

*Table 12: Items in facility rooms (Observation` and record review)*

*Do ask for any items that are there in stock register and are stored due to non-utilisation or non- functionality*

| ***Name of the equipment or furniture*** | ***Quantity of functioning items in each room*** | | | | | | | | | | | | | | | | |
| --- | --- | --- | --- | --- | --- | --- | --- | --- | --- | --- | --- | --- | --- | --- | --- | --- | --- |
|  | ***Room no. 1*** | ***Room no. 2*** | ***Room no. 3*** | ***Room no. 4*** | ***Room no. 5*** | ***Room no. 6*** | ***Room no. 7*** | ***Room no. 8*** | ***Room no. 9*** | ***Room no. 10*** | ***Room no. 11*** | ***Room no. 12*** | ***Room no. 13*** | ***Room no. 14*** | ***Room no. 15*** | ***Room no. 16*** | ***Corridors*** |
| Almirah (Big steel) |  |  |  |  |  |  |  |  |  |  |  |  |  |  |  |  |  |
| Almirah (Small steel) |  |  |  |  |  |  |  |  |  |  |  |  |  |  |  |  |  |
| Almirahs (Small wooden) |  |  |  |  |  |  |  |  |  |  |  |  |  |  |  |  |  |
| Armless chairs |  |  |  |  |  |  |  |  |  |  |  |  |  |  |  |  |  |
| Bed side attendant chair |  |  |  |  |  |  |  |  |  |  |  |  |  |  |  |  |  |
| Bed side locker |  |  |  |  |  |  |  |  |  |  |  |  |  |  |  |  |  |
| Bed side Screen |  |  |  |  |  |  |  |  |  |  |  |  |  |  |  |  |  |
| Bed side table |  |  |  |  |  |  |  |  |  |  |  |  |  |  |  |  |  |
| Buckets |  |  |  |  |  |  |  |  |  |  |  |  |  |  |  |  |  |
| Centrifuge |  |  |  |  |  |  |  |  |  |  |  |  |  |  |  |  |  |
| Bulbs |  |  |  |  |  |  |  |  |  |  |  |  |  |  |  |  |  |
| CFL tubes |  |  |  |  |  |  |  |  |  |  |  |  |  |  |  |  |  |
| Clock /watch |  |  |  |  |  |  |  |  |  |  |  |  |  |  |  |  |  |
| Coat rack |  |  |  |  |  |  |  |  |  |  |  |  |  |  |  |  |  |
| Curtain rods |  |  |  |  |  |  |  |  |  |  |  |  |  |  |  |  |  |
| Curtains |  |  |  |  |  |  |  |  |  |  |  |  |  |  |  |  |  |
| Cylinder |  |  |  |  |  |  |  |  |  |  |  |  |  |  |  |  |  |
| Delivery table |  |  |  |  |  |  |  |  |  |  |  |  |  |  |  |  |  |
| Dressing trolley |  |  |  |  |  |  |  |  |  |  |  |  |  |  |  |  |  |
| Drum with tap for storing water |  |  |  |  |  |  |  |  |  |  |  |  |  |  |  |  |  |
| Examination bed or table |  |  |  |  |  |  |  |  |  |  |  |  |  |  |  |  |  |
| Fans |  |  |  |  |  |  |  |  |  |  |  |  |  |  |  |  |  |
| Foot step |  |  |  |  |  |  |  |  |  |  |  |  |  |  |  |  |  |
| Hand washing basin |  |  |  |  |  |  |  |  |  |  |  |  |  |  |  |  |  |
| Height measuring stand |  |  |  |  |  |  |  |  |  |  |  |  |  |  |  |  |  |
| Inpatient iron bed |  |  |  |  |  |  |  |  |  |  |  |  |  |  |  |  |  |
| Kerosene stove |  |  |  |  |  |  |  |  |  |  |  |  |  |  |  |  |  |
| Labour table |  |  |  |  |  |  |  |  |  |  |  |  |  |  |  |  |  |
| Large medicine cupboard |  |  |  |  |  |  |  |  |  |  |  |  |  |  |  |  |  |
| Large steel benches |  |  |  |  |  |  |  |  |  |  |  |  |  |  |  |  |  |
| Large wooden benches |  |  |  |  |  |  |  |  |  |  |  |  |  |  |  |  |  |
| Mattress |  |  |  |  |  |  |  |  |  |  |  |  |  |  |  |  |  |
| Medicine chest |  |  |  |  |  |  |  |  |  |  |  |  |  |  |  |  |  |
| Medicine trolley |  |  |  |  |  |  |  |  |  |  |  |  |  |  |  |  |  |
| Metal chair |  |  |  |  |  |  |  |  |  |  |  |  |  |  |  |  |  |
| Metal file cabinet |  |  |  |  |  |  |  |  |  |  |  |  |  |  |  |  |  |
| Microscope |  |  |  |  |  |  |  |  |  |  |  |  |  |  |  |  |  |
| Mugs |  |  |  |  |  |  |  |  |  |  |  |  |  |  |  |  |  |
| Operation Lamp |  |  |  |  |  |  |  |  |  |  |  |  |  |  |  |  |  |
| Plastic bin |  |  |  |  |  |  |  |  |  |  |  |  |  |  |  |  |  |
| Refrigerator |  |  |  |  |  |  |  |  |  |  |  |  |  |  |  |  |  |
| Rubber / plastic shutting |  |  |  |  |  |  |  |  |  |  |  |  |  |  |  |  |  |
| Sauce pan with lid |  |  |  |  |  |  |  |  |  |  |  |  |  |  |  |  |  |
| Side Wall mounted fan |  |  |  |  |  |  |  |  |  |  |  |  |  |  |  |  |  |
| Side wooden racks |  |  |  |  |  |  |  |  |  |  |  |  |  |  |  |  |  |
| Sink |  |  |  |  |  |  |  |  |  |  |  |  |  |  |  |  |  |
| Stool (steel ) |  |  |  |  |  |  |  |  |  |  |  |  |  |  |  |  |  |
| Stool (wooden) |  |  |  |  |  |  |  |  |  |  |  |  |  |  |  |  |  |
| stove 2 burner |  |  |  |  |  |  |  |  |  |  |  |  |  |  |  |  |  |
| Stretcher |  |  |  |  |  |  |  |  |  |  |  |  |  |  |  |  |  |
| Swab rack |  |  |  |  |  |  |  |  |  |  |  |  |  |  |  |  |  |
| Telephone |  |  |  |  |  |  |  |  |  |  |  |  |  |  |  |  |  |
| Three seater steel chairs |  |  |  |  |  |  |  |  |  |  |  |  |  |  |  |  |  |
|  |  |  |  |  |  |  |  |  |  |  |  |  |  |  |  |  |  |
|  |  |  |  |  |  |  |  |  |  |  |  |  |  |  |  |  |  |
|  |  |  |  |  |  |  |  |  |  |  |  |  |  |  |  |  |  |

**Section 13: Details about IEC material**

*Table 13: Signage/ IEC material on display in the unit (Observation cum record review in stock register)*

| **Type of IEC material (Specify size)** | ***Quantity*** | ***Expenditure*** | ***Utility (OPD=1, IPD=2, Out-reach=3,OPD+IPD=4, All= 5, OPD+OR=6, IPD+ OR=7)*** | ***List services for which it is used. Write serial number codes from section 21*** |
| --- | --- | --- | --- | --- |
| Flex board |  |  |  |  |
| Paper Charts |  |  |  |  |
| Wall paintings |  |  |  |  |
|  |  |  |  |  |
|  |  |  |  |  |
|  |  |  |  |  |

**Section 14: Details of stationary items**

*Table 14: Stationary and other miscellaneous items: (Record review for billed amounts of purchased stationary)*

| **Item** | **Quantity** | ***Utility (OPD=1, IPD=2, Out-reach=3,OPD+IPD=4, All= 5, OPD+OR=6, IPD+ OR=7)*** | ***List services for which it is used. Write serial number codes from section 21*** |
| --- | --- | --- | --- |
| Article indent book |  |  |  |
| Attendance register |  |  |  |
| Bath soap |  |  |  |
| Carbon paper |  |  |  |
| Cash receipt book |  |  |  |
| Disinfectant fluids (Phenly-Ltr) |  |  |  |
| Harpic |  |  |  |
| Health management info system subcenter register |  |  |  |
| Indoor register |  |  |  |
| Nirma |  |  |  |
| OPD card |  |  |  |
| OPD register |  |  |  |
| Out-station dak book |  |  |  |
| Pencil |  |  |  |
| Broom |  |  |  |
| Photostat paper |  |  |  |
| Pocha |  |  |  |
| Poly bags for biowaste |  |  |  |
| Register IDSP |  |  |  |
| Savlon solution |  |  |  |
| Spirit |  |  |  |
| Stamp ink |  |  |  |
| Stamp pad |  |  |  |
| Stock + OPD register |  |  |  |
| Toilet brush |  |  |  |
| Towels + dusters |  |  |  |
| A-4 paper |  |  |  |
| Vim powder |  |  |  |
| Article indent book |  |  |  |
| Attendance register |  |  |  |
| Bath soap |  |  |  |
| Carbon paper |  |  |  |
| Cash receipt book |  |  |  |
| Disinfectant fluids (Phenly-Ltr) |  |  |  |
| Harpic |  |  |  |
| Health management info system register |  |  |  |
| Indoor register |  |  |  |
| Nirma |  |  |  |
| OPD card |  |  |  |
| OPD register |  |  |  |
| Out-station dak book |  |  |  |
| Pencil |  |  |  |
| Broom |  |  |  |
| Photostat paper |  |  |  |
| Pocha |  |  |  |
| Poly bags for biowaste |  |  |  |
| Register IDSP |  |  |  |
| Savlon solution |  |  |  |
| Spirit |  |  |  |
| Stamp ink |  |  |  |
| Stamp pad |  |  |  |
|  |  |  |  |
|  |  |  |  |

**Section 15: Utilities**

*Table 15: Utilities/ Overhead (Annual)*

|  | ***Quantity*** | ***Expenditure*** | ***Utility (OPD=1, IPD=2, Out-reach=3,OPD+IPD=4, All= 5, OPD+OR=6, IPD+ OR=7)*** | ***List services for which it is used. Write serial number codes from Tables 21*** |
| --- | --- | --- | --- | --- |
| ***1.Means of transport*** |  |  |  |  |
| Maintenance |  |  |  |  |
| Repairs |  |  |  |  |
| Insurance |  |  |  |  |
| Others |  |  |  |  |
| Total *(If available)* |  |  |  |  |
| ***2. Building*** |  |  |  |  |
| Electricity |  |  |  |  |
| Water |  |  |  |  |
| Facility rent (if relevant) |  |  |  |  |
| Maintenance |  |  |  |  |
| Telephone |  |  |  |  |
| Kerosene |  |  |  |  |
| Other |  |  |  |  |
| Total *(If available)* |  |  |  |  |
| ***3. Equipment*** |  |  |  |  |
| Maintenance |  |  |  |  |
| Repairs |  |  |  |  |
| Other |  |  |  |  |
| Total *(If available)* |  |  |  |  |
| ***4. Laundry*** |  |  |  |  |
|  |  |  |  |  |
|  |  |  |  |  |
|  |  |  |  |  |

**Section 16: Laboratory/ Radiological investigation/Procedure**

*Table 16: Laboratory/ Radiological investigation/Procedure*

| **Type of tests** | **Quantity** | ***Utility (OPD=1, IPD=2, Out-reach=3,OPD+IPD=4, All= 5, OPD+OR=6, IPD+ OR=7)*** | ***List services for which it is used. Write serial number codes from section 21*** |
| --- | --- | --- | --- |
| Haemoglobin |  |  |  |
| TLC |  |  |  |
| DLC |  |  |  |
| ESR |  |  |  |
| Malaria parasite |  |  |  |
| Sputum testing for TB (AFB) |  |  |  |
| Routine urine |  |  |  |
| Widal |  |  |  |
| Blood grouping |  |  |  |
| Bleeding time, clotting time (BT and CT |  |  |  |
| Cholesterol |  |  |  |
| Urea |  |  |  |
| Uric acid |  |  |  |
| Blood sugar |  |  |  |
| Rapid tests for pregnancy |  |  |  |
| RPR test for Syphills/YAWS surveillance (in high endemic area only) VDRL |  |  |  |
| Rapid tests for HIV |  |  |  |
| RA factor |  |  |  |
| **Diagnostic test (General)** |  |  |  |
| X-rays |  |  |  |
| Ultrasound |  |  |  |
|  |  |  |  |
|  |  |  |  |
|  |  |  |  |
|  |  |  |  |

| ***Diagnostic tests Dental*** | **Quantity** | ***Utility (OPD=1, IPD=2, Out-reach=3,OPD+IPD=4, All= 5, OPD+OR=6, IPD+ OR=7)*** | ***List services for which it is used. Write serial number codes from section 21*** |
| --- | --- | --- | --- |
| ***IOPA X-ray*** |  |  |  |
|  |  |  |  |
|  |  |  |  |
|  |  |  |  |

**Section 17: Details of referral transport**

*Table 17: Referral transport (Data to be taken from referral reports.*

| **Total number of patients referred from facility using referral transport** | **No. of Under-Fives** | **No. of Over-Fives** | ***List services for which it is used. Write serial number codes from section 21*** |
| --- | --- | --- | --- |
|  |  |  |  |
|  |  |  |  |
|  |  |  |  |
|  |  |  |  |

**Section 18: Details about cash benefits paid to patients**

*Table 18: Cash benefits paid to patients*

| **Name of Scheme** | **Amount paid during the period 2012-13** |
| --- | --- |
| JSY |  |
| Any other |  |
|  |  |
|  |  |
|  |  |

**Section 19: Details about utilisation of funds and grants**

*Table 19: Utilisation of funds and grants*

| **Funds and grants** | **Amount spent in the 2012-13** | ***List services for which it is used. Write serial number codes from section 21*** |
| --- | --- | --- |
| Alternate vaccine delivery in other areas |  |  |
| Annual Maintenance Grant for PHCs |  |  |
| ASHA incentive for completing 3 ANCs |  |  |
| Breast feeding-BCC/IEC |  |  |
| Incentive to ASHAs |  |  |
| Incentive to ASHA under child health |  |  |
| Infant death audit |  |  |
| Institutional deliveries |  |  |
| Jacha Bacha scheme |  |  |
| JSSK |  |  |
| Maternal death review/audit |  |  |
| Measles campaign grant |  |  |
| Mobility support for school health officer |  |  |
| Mobility support for supervision for state & district level |  |  |
| Mobilization of children through ASHA or other mobilizers |  |  |
| Monitoring & supervision of IMNCI activities |  |  |
| Monthly meeting of PEEs-ARSH |  |  |
| Other strategies/activities (family planning) |  |  |
| Performance incentive/other incentive to ASHAs |  |  |
| RCH outreach camps |  |  |
| Supervisory visit-BCC/IEC |  |  |
| Untied fund for PHCs |  |  |
| Untied fund for VHSC (PHC level) |  |  |
|  |  |  |
|  |  |  |

**Section 20: Morbidity profile**

*Table 20a: Morbidity profile of patients treated in out-patient department (OPD) from April 2014-March 2012-13 (If not available for the whole year, collect data for specific months of April 2014, August 2014 and January 2012-13)*

| **S.No.** | **Morbidity** | **Number of Patients** | **Time duration*(if not annual)** | **Period of data collected** |
| --- | --- | --- | --- | --- |
|  |  |  |  |  |
|  |  |  |  |  |
|  |  |  |  |  |
|  |  |  |  |  |
|  |  |  |  |  |
|  |  |  |  |  |
|  |  |  |  |  |
|  |  |  |  |  |
|  |  |  |  |  |
|  |  |  |  |  |
|  |  |  |  |  |
|  |  |  |  |  |
|  |  |  |  |  |
|  |  |  |  |  |
|  |  |  |  |  |
|  |  |  |  |  |
|  |  |  |  |  |
|  |  |  |  |  |
|  |  |  |  |  |
|  |  |  |  |  |
|  |  |  |  |  |
|  |  |  |  |  |
|  |  |  |  |  |
|  |  |  |  |  |
|  |  |  |  |  |

****If the data is not available for the last one year then mention the time duration for the recorded data. For example: 1 month, 2 months, 6 months etc.***

***Table 20b: Morbidity profile of patients hospitalized (IPD) from April 2014-March 2012-13***

| **S.No.** | **Morbidity** | **Number of Patients** | **Time duration*(if not annual)** | **Period of data collected** |
| --- | --- | --- | --- | --- |
|  |  |  |  |  |
|  |  |  |  |  |
|  |  |  |  |  |
|  |  |  |  |  |
|  |  |  |  |  |
|  |  |  |  |  |
|  |  |  |  |  |
|  |  |  |  |  |
|  |  |  |  |  |
|  |  |  |  |  |
|  |  |  |  |  |
|  |  |  |  |  |
|  |  |  |  |  |
|  |  |  |  |  |
|  |  |  |  |  |
|  |  |  |  |  |
|  |  |  |  |  |
|  |  |  |  |  |
|  |  |  |  |  |
|  |  |  |  |  |
|  |  |  |  |  |
|  |  |  |  |  |
|  |  |  |  |  |
|  |  |  |  |  |
|  |  |  |  |  |
|  |  |  |  |  |

**Section 21: Time allocation sheet#**

*Table 21: Staff Member Code (Enter Code as entered in Table 2): ......*......................................

| **Service code no** | **Activity name** | **Type of activity** | | **Fixed schedule activity** | | | **Routine activity** | | |
| --- | --- | --- | --- | --- | --- | --- | --- | --- | --- |
|  |  | **Fixed schedule** | **Routine** | **Frequency (once in a week/once in month/twice a week etc.)*** | **Hours per day of activity** | **Days for which the activity was done during the year 2012-13** | **Time per person (in minutes) (a)** | **Number of beneficiaries on a routine day (b)** | **If not (a) and (b) then how much time spent to do the activity** |
|  | *Ante natal care (HB estimation, BP, TT, Physical Exam, IFA)* |  |  |  |  |  |  |  |  |
|  | *Institutional deliveries* |  |  |  |  |  |  |  |  |
|  | *Post natal care* |  |  |  |  |  |  |  |  |
|  | *New born care corner* |  |  |  |  |  |  |  |  |
|  | *Immunisation* |  |  |  |  |  |  |  |  |
|  | *Routine OPD (over 5)* |  |  |  |  |  |  |  |  |
|  | *Routine OPD (U-5 )* |  |  |  |  |  |  |  |  |
|  | *Family Planning: Tubectomy motivation* |  |  |  |  |  |  |  |  |
|  | *Tubectomy procedure* |  |  |  |  |  |  |  |  |
|  | *Family Planning: IUCD motivation* |  |  |  |  |  |  |  |  |
|  | *IUCD procedure* |  |  |  |  |  |  |  |  |
|  | *Family planning: Counselling Oral pill and Condom distribution* |  |  |  |  |  |  |  |  |
|  | *Special day care services*   - *Primary management wounds* - *Primary management fracture* - *Primary management abscess drainage* - *Primary management burns* |  |  |  |  |  |  |  |  |
|  | *IPD care for management of cases related to Medicine (Malaria, Dengue, Typhoid, simple fever, dog and snake bites, poisonings, burn, pneumonia, dehydration, etc)* |  |  |  |  |  |  |  |  |
|  | *IPD: Management of Accidental cases* |  |  |  |  |  |  |  |  |
|  | *IPD: General rounds* |  |  |  |  |  |  |  |  |
|  | *Operation theatre* |  |  |  |  |  |  |  |  |
|  | *Dental procedures* |  |  |  |  |  |  |  |  |
|  | *Emergency duty* |  |  |  |  |  |  |  |  |
|  | *AYUSH services (OPD)* |  |  |  |  |  |  |  |  |
|  | *DOTS provision* |  |  |  |  |  |  |  |  |
|  | *Outreach services (like vaccination, surveillance, etc.)* |  |  |  |  |  |  |  |  |
|  | *School health programs* |  |  |  |  |  |  |  |  |
|  | *Adolescent health programs* |  |  |  |  |  |  |  |  |
|  | *Any other health programs (National health programs)* |  |  |  |  |  |  |  |  |
|  | *Special duties (Like during emergencies, jails, etc.)* |  |  |  |  |  |  |  |  |
|  | *Posting to another facility* |  |  |  |  |  |  |  |  |
|  | *Meetings at district level* |  |  |  |  |  | **NA** | **NA** |  |
|  | *Meetings at the state head quarters* |  |  |  |  |  | **NA** | **NA** |  |
|  | *Meetings in CHC* |  |  |  |  |  | **NA** | **NA** |  |
|  | *Meetings at PHC itself* |  |  |  |  |  | **NA** | **NA** |  |
|  | *Meeting with ANM’s from Sub centre* |  |  |  |  |  | **NA** | **NA** |  |
|  | *Meetings with local bodies* |  |  |  |  |  | **NA** | **NA** |  |
|  | *Any other meetings* |  |  |  |  |  | **NA** | **NA** |  |
|  | *Meetings with ASHAs* |  |  |  |  |  | **NA** | **NA** |  |
|  | *Routine administrative work* |  |  |  |  |  | **NA** | **NA** |  |
|  | *Family planning camp* |  |  |  |  |  |  |  |  |
|  | *Health camps for treatment of minor ailments* |  |  |  |  |  |  |  |  |
|  | *Other health camps* |  |  |  |  |  |  |  |  |
|  | *Pulse polio immunisation* |  |  |  |  |  |  |  |  |
|  | *Village Health and Nutrition days* |  |  |  |  |  | **NA** | **NA** |  |
|  | *IEC Activities related to National Health programmes* |  |  |  |  |  | **NA** | **NA** |  |
|  | *Disease surveillance (Early detection of cases (as per IDSP guidelines)and Control of local endemic diseases* |  |  |  |  |  | **NA** | **NA** |  |
|  | *Maintenance of record, registers and reports (routine HMIS)* |  |  |  |  |  | **NA** | **NA** |  |
|  | *House to house surveys* |  |  |  |  |  |  |  |  |
|  | *Water and Sanitation(Disinfection of drinking water source , Promotion of sanitation)* |  |  |  |  |  | **NA** | **NA** |  |
|  | *Monitoring and supervision activities* |  |  |  |  |  | **NA** | **NA** |  |
|  | *Trainings conducted for staff at the facility* |  |  |  |  |  | **NA** | **NA** |  |
|  | *Support activities for all direct services Like cleanliness, sweeping, clerical work* |  |  |  |  |  | **NA** | **NA** |  |
|  | *Ambulatory services* |  |  |  |  |  |  |  |  |

****‘1’ for once a year*** *participation, ‘****2’ for twice a year****,* ***3 for thrice a year*** *participation,* ***4 for quarterly participation****,* ***5 for once every two months****,* ***6 for monthly participation****,* ***7 for fortnightly participation****,* ***8 for weekly participation****,* ***9 for twice a week participation****,* ***10 for thrice a week*** *participation.*

***#This information should be taken from all the MO’s posted at PHC and one staff nurse per OPD, IPD, ward etc.***
